# Knowledge of Antibiotic Management in Surgery, Periodontics and Endodontics Among Patients, Students and Dentistry Professors: A Cross-Sectional Study at the University of Barcelona (Spain)

**DOI:** 10.3390/jcm14072179

**Published:** 2025-03-22

**Authors:** Adrián Toribio-Méndez, Paloma Montero-Miralles, Sonia Egido-Moreno, Verónica Schiavo Di Flaviano, Beatriz González-Navarro, José López-López

**Affiliations:** 1 Department of Odontostomatology, Faculty of Medicine and Health Sciences (Dentistry), University of Barcelona, L’Hospitalet de Llobregat, 08970 Barcelona, Spain; adri7toribio@gmail.com (A.T.-M.); v.schiavo@ub.edu (V.S.D.F.); beatrizgonzalez@ub.edu (B.G.-N.); 2 Department of Stomatology (Endodontic Section), School of Dentistry, University of Sevilla, C/Avicena s/n, 41009 Sevilla, Spain; montero_paloma@hotmail.com; 3 Oral Health and Masticatory System 19 Group, Institut d’Investigació Biomédica de Bellvitge IDIBELL (Bellvitge Institute of Biomedical Research), 08907 Barcelona, Spain

**Keywords:** antibiotics, antibiotics and dentistry, antibiotic resistance

## Abstract

**Background/Objectives**: The main objective of this study was to analyze the knowledge on the use and management of antibiotics in dentistry within three different groups of interest: patients, fifth-year dental students at the University of Barcelona and professors of the faculty of dentistry of the University of Barcelona. **Methods**: A cross-sectional pilot study was carried out using questionnaires addressed to the three groups of interest: patients (*n* = 250), students (*n* = 79) and professors (*n* = 50). Sociodemographic questions were asked of all the groups. The professor and student questions were related to antibiotic therapy in relation to dental procedures. The patients were asked questions related to antibiotic management. **Results**: Regarding the patient group, there were statistically significant differences between the participants of the group; people without higher education were more likely to self-medicate (*p* = 0.043) or to be unaware of the adverse effects (*p* = 0.045). Regarding the student and professor groups, there were no significant differences in the prescription of antibiotics. Amoxicillin 750 mg was the most commonly used in patients without an allergy to Penicillin, but there were significant differences in the antibiotic of choice for those patients allergic to Penicillin, the most commonly prescribed being either Clindamycin or Azithromycin (*p* = 0.002). **Conclusions**: The study revealed a lack of uniformity in the knowledge and management of antibiotics among both students and professors, which highlights the need to improve university training in pharmacology and for professors to continue education throughout their working lives. It also indicates the need for patient health education regarding antibiotics.

## 1. Introduction

An antibiotic is understood as any bactericidal substance, regardless of its origin, whether natural, synthetic or semi-synthetic [1]. In 1928, Alexander Fleming accidentally discovered penicillin. Thanks to the discovery and use of antibiotics, human life expectancy has increased by an average of 23 years [1,2].

According to the World Health Organization (WHO), antibiotics are the most misused drugs due to their easy access, low cost, familiarity and wide safety margin. This widespread use has led to antibiotic resistance, causing a global threat that could cause the loss of antibiotic effectiveness [3].

The oral microbiome is one of the most complex bacterial communities in the human body. Numerous infectious diseases occur in the mouth, whether of dental origin, such as caries, or periodontal infections, for example, gingivitis or periodontitis [4,5,6].

As dentists prescribe approximately 10% of antibiotics dispensed in primary care, it is important not to underestimate the potential contribution of the dental profession to the development of antibiotic-resistant bacteria [7]. Currently, antibiotic-resistant bacteria are a global health problem since they increase the biological risk for patients (increased morbidity and mortality rates) and constitute an economic burden for national healthcare systems [2]. In a paper recently published by The Lancet, it was estimated that in 2021, 4.71 million deaths were associated with antibiotic-resistant bacteria [8].

The incorrect prescription of antibiotics for pathologies such as irreversible pulpitis, pulp necrosis and acute localized periodontitis, and as pain relief in irreversible pulpitis, inadequate dosage and excessive duration of treatment are some of the factors that have been studied in previous papers [9,10,11] and may be associated with the development of bacterial resistance to antibiotics. Another factor influencing the inappropriate use of antibiotics involves social policies such as a lack of control in their sale or pressure exerted by the patients that can lead to the dentist inappropriately prescribing antibiotics to please the patient [12]. Insufficient knowledge of the correct indications for antibiotic prescription or the patient’s belief that they need antibiotics to prevent complications has led to overprescription and self-medication [13]. For these reasons, the implementation of educational strategies to increase awareness of correct antibiotic use [13] and modify the dental curriculum are required to improve dental student knowledge about the coherent and proper use of antibiotics [9].

Regarding antibiotic resistance, efforts are being made to find new substances to combat this growing problem. Different research groups are trying to find new compounds, such as darobactin, with antibacterial capabilities to curb the resistance crisis [14]. Interesting advances are also being made in AI research, particularly in the discovery of new antibiotics using deep learning models. AI-driven methods help identify novel antimicrobial compounds, predict bacterial resistance, and optimize antibiotic prescription. These approaches accelerate drug research and improve our understanding of resistance mechanisms. Additionally, AI contributes to reducing antibiotic misuse by analyzing large datasets and enhancing treatment strategies [15].

There are some other articles that evaluate the knowledge dental students, practitioners and patients have about the management of antibiotics of [10,12,13,16]. However, with this study, we aim to analyze the differences in the knowledge on the use and management of antibiotics in dentistry and between three different groups of interest: patients, fifth-year dental students at the University of Barcelona and professors of the faculty of dentistry of the University of Barcelona.

The study aims to assess the knowledge of the proper use of antibiotics among various groups—patients, dental students and professors—to identify possible areas for improvement. It is hypothesized that, in general, patients lack sufficient knowledge about the proper use of antibiotics and that older patients, as well as those without higher education, are less knowledgeable in particular. Additionally, dental students demonstrate less knowledge about proper antibiotic prescribing, dosing and duration compared to professors. Professors with a specialty are also expected to be more knowledgeable regarding the appropriate use of antibiotics than non-specialists. The objectives of this study aim to highlight disparities in antibiotic knowledge and support strategies to improve education and awareness.

## 2. Materials and Methods

### 2.1. Study Design

A cross-sectional pilot study was carried out using two questionnaires: one for professors who teach at the Dental Hospital of the University of Barcelona (HOUB) and fifth-year dentistry students at the University of Barcelona (UB) (Dentistry Degree) and another for HOUB patients. See Supplementary Materials.

The study followed the Strengthening the Reporting of Observational Studies in Epidemiology (STROBE) statement [17].

The study protocol was approved on 19 February 2024, with the identification number (10-2024), by the Committee on Ethics and Research with Medicines (CEIm HOUB) of the city of Barcelona, Spain. The Declaration of Helsinki guidelines on research involving human subjects were followed throughout the study.

### 2.2. Questionnaires

The questionnaires were based on those formulated in previous surveys in Seville (Spain) [10,12] with some modifications. Both questionnaires requested sociodemographic data. In the case of professors and students, 14 questions were asked, such as the type of antibiotic choice, prescription duration, antibiotic prophylaxis and clinical situations that might require antibiotic administration. In the case of patients, 25 questions were asked related to the possible dental treatments that had been performed, their beliefs in relation to the prescription of antibiotics after or before different treatments and situations and the benefits or detriments of taking antibiotics, among other issues. Professors of HOUB who were not part of answering the questionnaires reviewed the questions for appropriateness and clarity. The patients who participated in the survey did so anonymously, voluntarily and without compensation.

### 2.3. Data Source, Participant Identification and Recruitment

All the participants signed an informed consent and were of legal age (18 years old). All the participants were recruited at the HOUB. The patients who attended were surveyed at the entrance to and exit of the waiting rooms. The professors participated during their teaching activity. The students participated during their internships. All questionnaires were collected during the months of February and March of 2024, and once they were collected, data analysis was performed.

### 2.4. Selection Criteria

To be considered a patient in the study, the participant had to be over 18 years of age and attend the HOUB. In addition, they had to accept and understand the conditions of the study and sign the informed consent form. It is important to note that the patients did not have to have a degree in dentistry or have completed studies related to the health area. The students who were eligible to participate had to be fifth-year dental students at the University of Barcelona. Like the patients, they had to be over 18 years of age and accept and understand their participation in the study by signing the informed consent form. The participating professors had to be undergraduate and/or postgraduate professors at HOUB. They also had to accept and understand the conditions of the study and sign the informed consent to participate.

### 2.5. Masking Techniques

The groups were coded as follows: Group 1: patients, Group 2: fifth-year students, and Group 3: professors. Anonymization was performed as follows: No data that could reveal the person’s identity was included in the questionnaires. These questionnaires were filed in closed, opaque containers, which were opened once the data collection process was completed. The containers were opened at the end of the questionnaire collection in the presence of two witnesses not involved in the study to demonstrate that the container had not been tampered with before opening and that the questionnaires were blinded to those investigated until the end of the process.

In completing the questionnaire, no name or personal email address was requested to control anonymity. The only data requested from the patients were: (Group 1) gender, age range and educational level; (Group 2, Group 3) gender, age range, specialty and length of practice in the profession for professors and students; (Group 1, 2, 3) answers to the questions on the questionnaire.

### 2.6. Sample Size

Although we proposed a pilot study, we performed a sample calculation based on the sample calculation formula for finite populations. n = N × Z2p × q/e2(N − 1) + Z2 × p × q. n: size of the sample sought; N: size of the universe or population; Z: level of confidence; e: error of estimation of the maximum accepted; p: probability of the event studied occurring; q: (1 × p) probability of the event studied not occurring.

For the study, we considered a confidence level of 95% and a margin of error of 5%. The number of patients who visited HOUB between February and March of 2024 was 1000; the number of fifth-year students was 100; and the number of HOUB professors was 50. The minimum expected sample size was 212, 74 and 43 patients, students and professors, respectively. We also found this estimate to be appropriate because it would allow us to compare it with previous studies.

### 2.7. Statistical Analysis

All the analyses were performed using SPSS statistical software, version 29.0.2.0 for Windows (SPSS, Chicago, IL, USA). Quantitative variables were described by frequency and percentage; qualitative variables were described, according to their distribution, as mean and standard deviation or median. Bivariate associations between categorical variables were assessed with the Fisher test for dichotomous variables and the Chi-square test for polychotomous variables. A *p*-value < 0.05 was considered statistically significant.

The statistical analysis was facilitated by carrying it out jointly for both the student and professor groups. The variables of “Having a specialty” were also regrouped into groups of related specialties to facilitate the statistical analysis.

## 3. Results

### 3.1. Demographic Data

Of the 250 patients surveyed in Group 1, 246 were valid, and the remaining four were discarded due to inconsistencies in the responses.

Female respondents (*n* = 131) accounted for 53.3% and males (*n* = 114) for 46.3%, while only one (0.4%) participant chose the gender “Other”. The most dominant age range was 65–85 years (26.5%), followed by 55–65 years (23.2%). The educational level of the patient participants was very heterogeneous, with higher levels of education being more prevalent than those with primary or general Basic education (23.6%) followed by high school (16.3%), as seen in Table 1.

Group 2 was composed of 79 fifth-year dentistry students. Of the 79 respondents, women (*n* = 61) represented 77.2% and men (*n* = 18) 22.80%. By far, the most prevalent age range was 20 to 25 years (67.1%), followed by 25 to 30 years (25.3%). In Group 3, 50 professors were surveyed, of whom 27 were female (54%) and 23 were male (46%). As for the age of the participants, the most dominant range was 55–70 years (28%), followed by 30–35 and 45–55 years, the latter was 25–30 years (5.3%) (Table 2).

### 3.2. Questionnaire Results

We present here the most significant results in a graphical manner. All the data related to the questionnaire can be reviewed in Supplementary Materials Tables S1–S8.

#### 3.2.1. Group 1: Patients

The variable “Other” in gender was excluded, and the variables “Age” and “Educational level” were recoded to facilitate statistical analysis in all bivariate data analyses.

Regarding the question of whether they had undergone endodontics, according to the age of the patients (*n* = 246), the lower ranges, between 18–35 years and 35–45 years, are those who have undergone this procedure the least, 24 (46.2%) and 22 (64.7%), respectively. On the other hand, of those over 65 years of age, 52 (80%) have undergone endodontics the most, with significant differences (*p* = 0.002).

Regarding the use of antibiotics after having undergone a root canal treatment, 58.1% of the total (*n* = 246) believe that it is not necessary, of whom 70 (61.4%) are men and 72 (55%) are women, with non-significant differences (*p* = 0.308). On the other hand, regarding the educational level, participants in the category “no studies” are those with the highest percentage believing that it is necessary (66.7%) compared to other educational levels, with significant differences (*p* = 0.031).

If the dentist says that they have a dental infection, the vast majority, 224 (91.4%) of respondents (*n* = 246), expect them to prescribe antibiotics, regardless of gender, with non-significant differences (*p* = 0.917). Age is not a determining factor either, with non-significant differences (*p* = 0.710). On the other hand, according to the educational level, the “no studies” respondents are those with the highest percentage (66.7%) who expect to be prescribed antibiotics, but this is not significant (*p* = 0.566).

If the professional did not prescribe antibiotics, the question is whether you would look for another professional and ask him/her why your doctor did not prescribe antibiotics. Regardless of age, gender and educational level, the vast majority of total respondents (*n* = 246), 174 (70.7%) answered “No”. On the other hand, 53 (21.5%) answered, “Yes, I would look for another dentist and ask him or her why my doctor has not prescribed antibiotics”.

**Figure 1:**
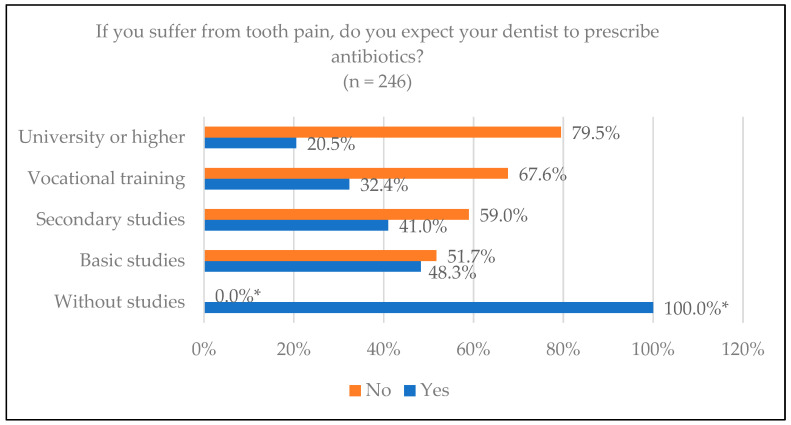
Patients who believe they should take antibiotics for dental pain according to their educational level. * Statistically significant differences.

Concerning the prescription of antibiotics in case of dental pain, of the total number of men (*n* = 114), 52 (45.6%) expected the prescription, compared to 41 (31.3%) women (*n* = 131) with significant differences (*p* = 0.021). It can be observed in Figure 1 that, as the educational level of the respondents increases, the percentage of those expecting a prescription decreases, ranging from 100% in the level categorized as no studies (*n* = 3) up to 20.5% of the group categorized as university or higher (being the highest educational level), with significant differences (*p* = 0.008).

**Figure 2:**
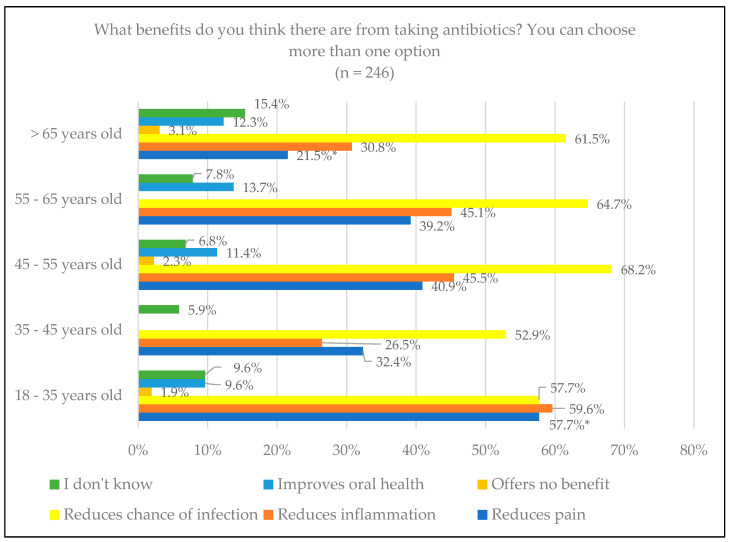
The expected benefits of antibiotics from patient knowledge by age. * Statistically significant differences.

**Figure 3:**
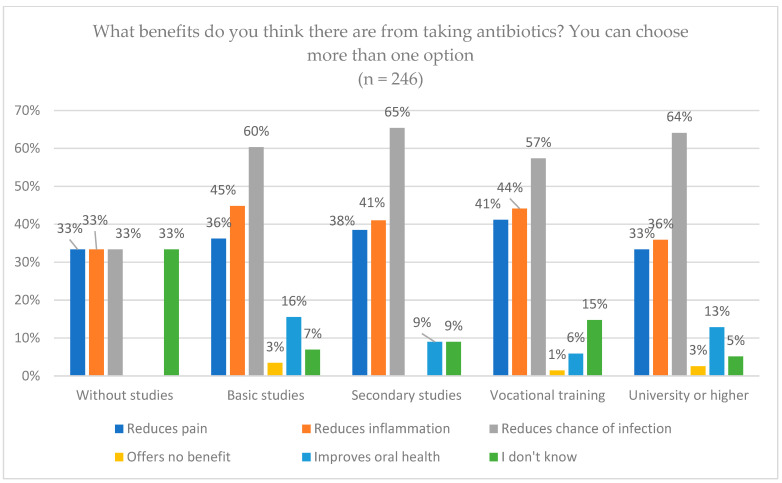
The expected benefits of antibiotics from patient knowledge by educational level.

Regarding the benefits of taking antibiotics, pain reduction was the variable with the greatest difference, and the reduction in the possibility of infection was the most selected by almost all the age groups, as shown in Figure 2. In the 18–35 age range, 57.7% chose this variable, compared to 21.5% of those over 65, with significant differences (*p* = 0.002). Regarding educational level, in terms of reducing the possibility of infection, only 33.3% of the uneducated group chose this option, the lowest percentage compared to the other groups, with no significant differences (*p* = 0.711) (Figure 3).

**Figure 4:**
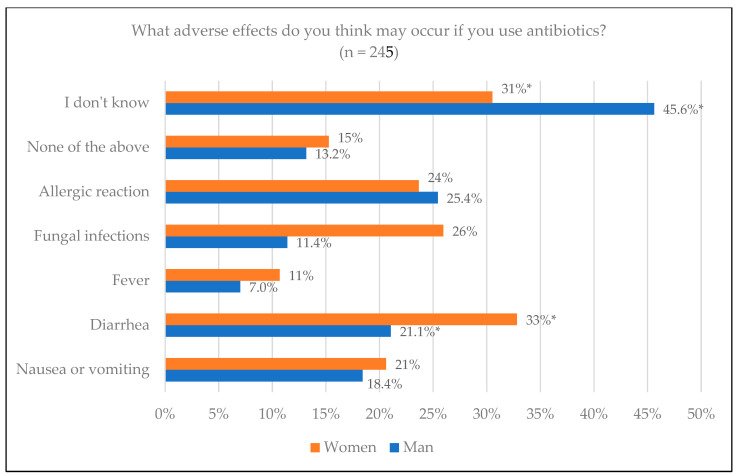
Adverse effects of antibiotics from patient knowledge according to sex. * Statistically significant differences.

Regarding perceptions of adverse effects that antibiotics can cause, we analyzed the responses of 245 participants, excluding the gender “Other” due to its low representation. Fifty-two (45.6%) of men out of the total (*n* = 114) marked the option “I don’t know”, the most chosen option for the gender; while in the case of women, 40 (30.5%) out of the total (*n* = 131) chosen this option, with significant differences (*p* = 0.015) (Figure 4). The diarrhea option was, in this case, the most chosen by women, specifically by 43 (32.8%) of those surveyed, as opposed to 24 (21.1%) men, with significant differences (*p* = 0.039).

**Figure 5:**
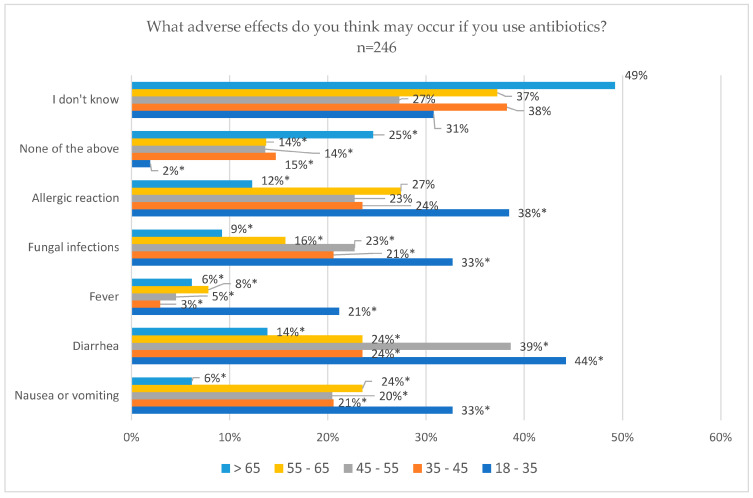
Adverse effects of antibiotics from patient knowledge according to age. * Statistically significant differences.

Considering age, the range of “over 65” was the one that chose the option “I don’t know” the most. Meanwhile, the youngest, 18–35 years, were the ones who marked the most specific adverse effects: nausea or vomiting 32.7% (*p* = 0.009), diarrhea 44.2% (*p* = 0.001), fever 21.1% (*p* = 0.012), fungal infections 32.7% (*p* = 0.028) and allergic reaction 38.5% (*p* = 0.026) (Figure 5).

**Figure 6:**
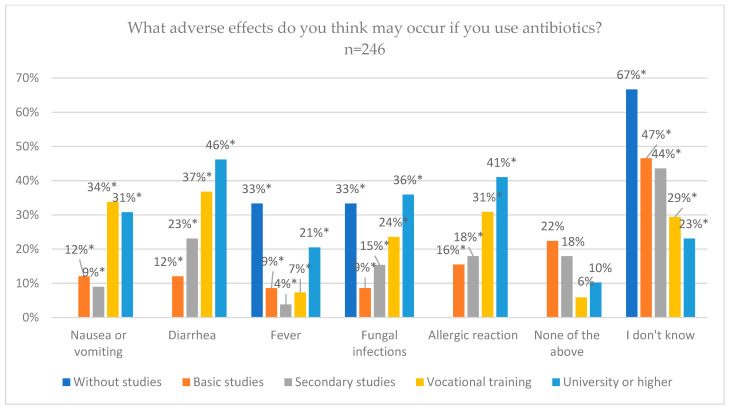
Adverse effects of antibiotics from patient knowledge according to educational level. * Statistically significant differences.

Concerning the educational level, the group with no studies and primary studies were the ones that answered “I don’t know” the most, 66.7% and 46.6%, respectively, with significant differences (*p* = 0.045). On the other hand, the group with university or higher studies was the one that chose the options of diarrhea 46.2% (*p* = 0.001), fungal infections 35.9% (*p* = 0.011) and allergic reaction 41% (*p* = 0.013) in greater proportions, with significant differences (Figure 6).

Regarding the knowledge of antibiotic resistance, of the 82 people who considered themselves knowledgeable and recognized that it is a topic of global health importance, 22 people (42.3%) were in the age range of 18–35 years, followed by 16 people (36.4%) in the next range, 45–55 years, with non-significant differences (*p* = 0.530). With regards to the educational level, 100% of the respondents in the group with no studies stated that they had no knowledge about antibiotic resistance, in contrast with 46.2% of the group with university studies who indicated that they had knowledge and believed that it was a topic of global importance, with significant differences (*p* = 0.012).

Regarding self-medication with antibiotics for toothache, a total of 66 (26.9%) of the respondents (*n* = 246) stated that they had self-medicated for toothache, and 72 (29.3%) had done so for a dental infection. On the other hand, 178 (73.2%) answered no for pain and 173 (70.6%) for an infection.

**Figure 7:**
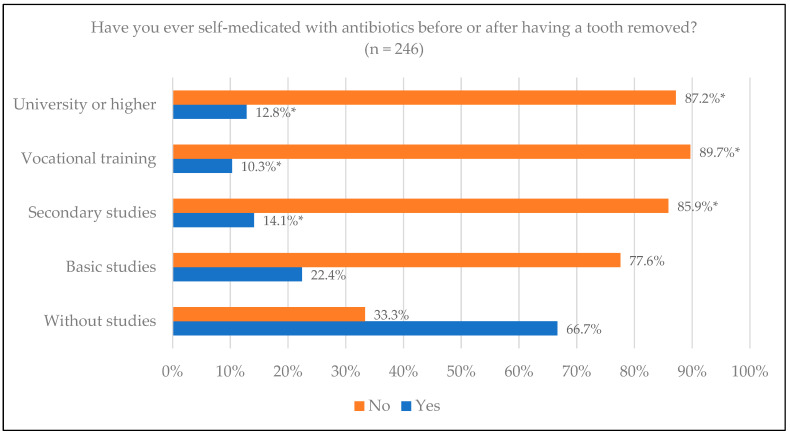
Having ever self-medicated with antibiotics before or after tooth extraction by educational level. * Statistically significant differences.

In relation to ever having self-medicated before or after having a tooth removed, of the total number of people surveyed (*n* = 246), the vast majority, 207 (84.5%), responded that they had not self-medicated. Of these, 61 (89.7%) belonged to the vocational training study group, with a higher proportion not having self-medicated together with university studies 34 (87.2%) and secondary studies 67 (85.9%), with significant differences (*p* = 0.043) (Figure 7).

#### 3.2.2. Group 2 and Group 3: Fifth-Year Dentistry Students and Professors

With regards to gender, of the total number of students (*n* = 79), 61 (77.22%) were women, with a similar proportion in postgraduate/master’s degree professors of 4 (80%), but being more heterogeneous in the category of undergraduate professor, where there were 13 (54.2%), and 11 undergraduate and in postgraduate/master’s degree professors (52.4%), with significant differences (*p* = 0.023).

**Figure 8:**
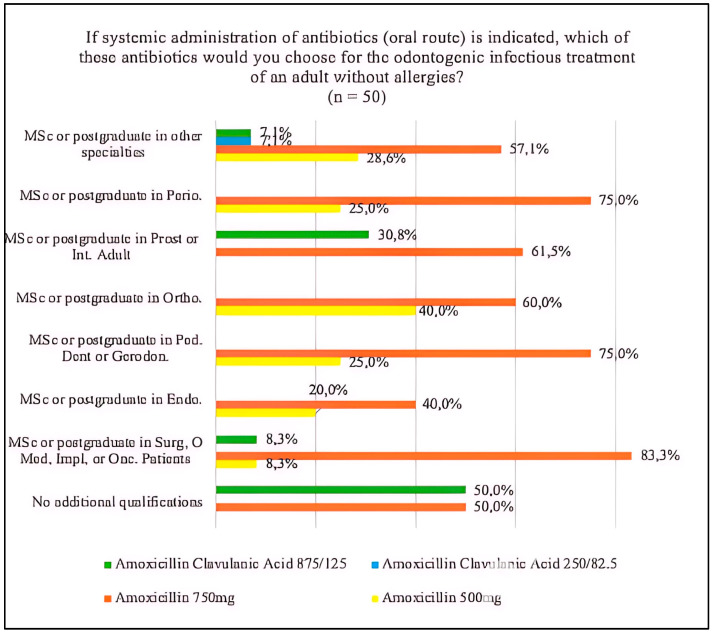
Drug choice in the systemic administration of antibiotics (oral route) in relation to specialty. Abbreviations in the figure: MSc (master’s), Postgraduate (pg), Perio. (periodontics), Prost. (prosthodontics), Int. Adult. (adult comprehensive dentistry), Ortho. (orthodontics), Ped. Dent. (pediatric dentistry), Gerodon. (gerodontology), Endo. (endodontics), Surg. (surgery), Med. (medicine), Impl. (implants), Onc. Patients (oncological patients).

In reference to the choice of antibiotic for the odontogenic infectious treatment of an adult without allergies, of the total number of professors surveyed (*n* = 50), more than half, 64%, chose Amoxicillin 750 mg. According to the specialty, a total of 10 (83.3%) of the professors of master’s or postgraduate degrees in surgery, oral medicine, implantology or oncological patients, and 2 (40%) professors of master’s or postgraduate degrees in endodontics chose Amoxicillin 750 mg, with non-significant differences (*p* = 0.433) [Figure 8]. Of the total number of students (*n* = 79), 63 (79.7%) chose Amoxicillin 750 mg and 11 (13.9%) Amoxicillin 500 mg. On the other hand, 8 (16%) of the professors chose Amoxicillin 500 mg and 6 (12%) Amoxicillin Clavulanic Acid 875/125, respectively, with non-significant differences (*p* = 0.062).

**Figure 9:**
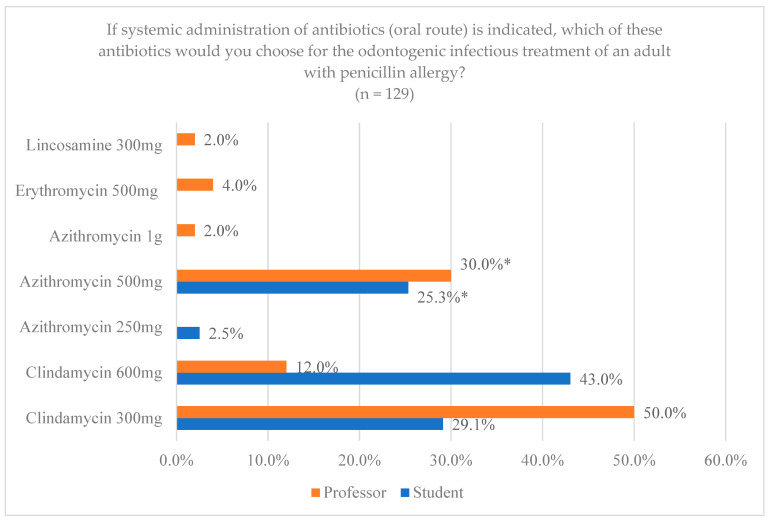
Drug choice by professors and students for the treatment of infections for adults with allergies. * Statistically significant differences.

For the duration of the antibiotic prescription, most of the professors (80%) chose 7 days, compared to 20% who chose 3–5 days. All (100%) of the professors without any additional qualification and those with a master’s or postgraduate degree in periodontology agreed with 7 days. On the other hand, 2 (50%) of the professors with a master’s or postgraduate degree in pediatric dentistry or gerodontology were those who, in greater proportion, chose 3–5 days, with non-significant differences (*p* = 0.324). In the case of the students, 72 (91.1%) chose 7 days, and 8.9% chose another option, with non-significant differences (*p* = 0.088). In the choice of drug for odontogenic infectious treatment for patients with a penicillin allergy, as shown in Figure 9, Clindamycin 600 mg was chosen by 34 (43%) students and 6 (12%) professors, and Clindamycin 300 mg by 23 (29.1%) students and 25 (50%) professors. On the other hand, Azithromycin 500 mg was chosen by 20 (25.3%) students and 15 (30%) professors, with significant differences (*p* = 0.002).

In relation to the specialty of the professors, Clindamycin 300 mg was chosen in greater proportion by the vast majority of specialties except by the professors of master’s or postgraduate studies in surgery, oral medicine, implantology or oncological patients and those of master’s or postgraduate studies in periodontology, 6 (50%) and 2 (50%), respectively, who selected Azithromycin 500 mg, with significant differences (*p* = 0.016).

With respect to the clinical situations for the prescription of antibiotics, 72 (91.1%) of the students and 46 (92%) of the professors indicated that they would prescribe the drug for “Pulp necrosis with symptomatic apical periodontitis, abscess, moderate or severe symptoms”, being the most chosen option by both groups, with non-significant differences (*p* = 0.865). Of 92% of the total number of professors (*n* = 50), 100% of each specialty chose this option, except in the case of professors with a master’s or postgraduate degree in prosthetics or integrated adult prosthetics, which numbered 12 (92.3%), with non-significant differences (*p* = 0.731).

**Figure 10:**
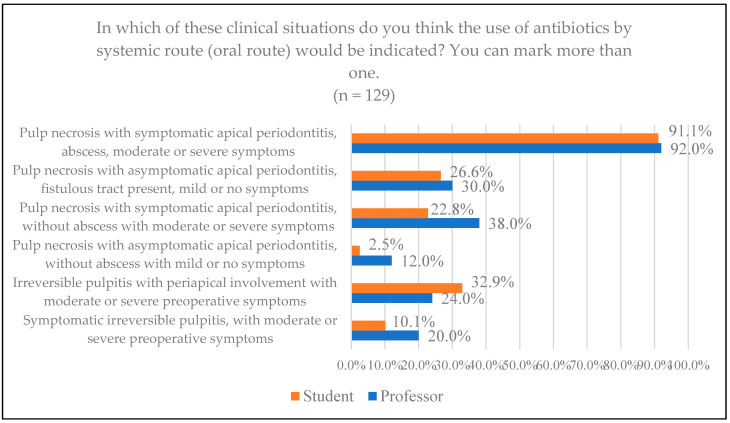
Situations chosen for the use of systemic antibiotics among professors and students.

Among the other options, the second most chosen by students was “Irreversible pulpitis with periapical involvement with moderate or severe preoperative symptoms”, of whom 26 (32.9%) students and 12 (24%) professors chose antibiotics, with non-significant differences (*p* = 0.279) (Figure 10). In the case of professors, the second most chosen option was “Symptomatic pulp necrosis, without abscess, with moderate or severe symptoms” by 19 (38%) of the professors and 18 (22.8%) of the students, with non-significant differences (*p* = 0.063) (Figure 10).

**Figure 11:**
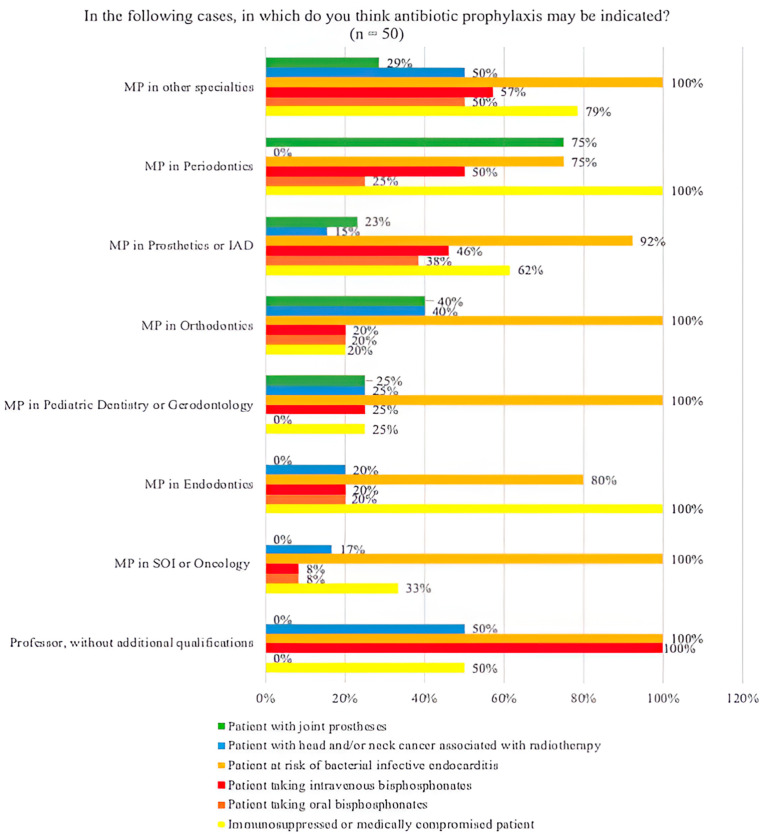
Bar chart of the possible cases in which antibiotic prophylaxis would be indicated according to the specialty. * Statistically significant differences; MP: master’s or postgraduate degree; IAD: integrated adult dentistry; SOI: surgery, oral medicine, implantology.

For the indication of antibiotic prophylaxis in clinical situations, “Patient at risk of bacterial endocarditis” was the most chosen option by most of the specialties, exceeding 80% in all, including professors without additional qualifications. The second most marked situation was “Patient immunosuppressed or medically compromised” by 60% of the professors. The least chosen was “Patient with joint prostheses” with 24%, with 0 (0%) professors without additional qualifications, a master’s or postgraduate degree in surgery, oral medicine, implantology or oncology patients and a master’s or postgraduate degree in endodontics, with significant differences (*p* = 0.002) (Figure 11).

## 4. Discussion

### 4.1. Key Results

This is the first study to investigate the knowledge and handling of three groups at once using two questionnaires, one for professors and students and another adapted for patients. The results obtained for the professor group could not be compared with other studies because they did not establish the same parameters as in previous works.

#### 4.1.1. Group 1: Patients

As for the patients having undergone a root canal treatment, participants in the lower age ranges, as expected, are those who have undergone this procedure the least, 46.2% of those in the 18–35 range and 64.7% of those in the 35–45 range, with those in the “Over 65” range being the ones who have undergone the procedure the most, with 80%. These results may be predictable since, with older age, a greater possibility of pulp pathology can be expected, related to the fact that, currently, the rate of tooth retention in older patients is higher than in years past [18].

Regarding the use of antibiotics after performing a root canal treatment, 58.1% of the total respondents (*n* = 246) believe that it is not necessary, compared to 41.9% who believe that it is, men (61.4%) representing a higher percentage than women (55%). These are similar results to other studies, in which 49.4% of the total respondents believe that it is necessary, and with a higher percentage of women (33%), unlike the study we present [12].

In reference to the prescription of antibiotics when there is a dental infection, 91.4% of respondents (*n* = 246) expected antibiotics to be prescribed, similar to other studies where it was 88.7% and 89.3 [12,13].

As to asking for a second professional opinion, 71% of respondents would not ask for a second opinion compared to 8% who would look for another dentist and would not ask why the previous one did not prescribe antibiotics, and 22% who would look for another dentist and ask why the previous one did not prescribe antibiotics. Therefore, 30% would seek a second opinion, compared to that in previous works, which showed 18% [13]. The result of this study shows that there is a certain distrust towards the medical care received.

Regarding dental pain, men (45.6%) more frequently expect the prescription of antibiotics than women (31.3%), in contrast with another study that found no differences by sex [12]. Of the respondents, 93 (37.8%) of the total (*n* = 246) indicated that they did expect the prescription, which is different from other studies, in which 53% of the total respondents expected the prescription for toothache [13]. It is necessary to explain and give written guidelines about the natural evolution after treatment to the patients so that they can better understand the indications of the drugs prescribed.

With regards to self-medication, 26.9% of the total respondents (*n* = 246) medicated themselves for toothache and 29.3% for infection. In other studies, 17.4% of the total respondents had self-medicated at some point. They also found differences in self-medication according to sex, with the majority being men, which was different from our study where we found no differences [12]. Regarding self-medication before or after removing a tooth, 84.5% responded that they did not do so; analyzing according to educational level, those who belonged to the vocational training studies group are the ones who self-medicated the least (89.7%), followed by those who had undertaken university studies (87.2%). In the systematic review on self-medication with antibiotics by Ahmed et al., 2023 [19], the researchers concluded that determinants related to the patient, as well as age, sex and cultural beliefs, among others, are associated with self-medication with antibiotics. Two studies, conducted in Eritrea and Georgia, conclude that self-medication with antibiotics is a major problem that contributes significantly to the development of antibacterial resistance when people use antibiotics without a prescription, often based on previous experiences or recommendations from friends or family, or by using leftover antibiotics [16,20]. As early as 2009, a study in our region showed how easy it was to obtain an antibiotic without a prescription [21]. Another Spanish study from 2017, with study variables similar to ours, such as gender, educational level and age, found that self-medication had increased based on studies by the same authors from previous years [22]. Although the trends in some regions, such as northern Europe and North America, seem to be downward, in Asia they are still very high [23].

To assess patients’ knowledge about antibiotics, they were asked about benefits and adverse effects. Reducing the chance of infection was the most chosen option by 61.4% of the total participants (*n* = 246), compared to previous studies, where in one it was 45.8% and 69.8% in another [10,13]. The age range of younger participants (18–35 years) was the one where most chose this option, different from another study where the age range identified the ones over 40 years old [12]. Regarding the perception of adverse effects that antibiotics can cause, 37.4% of the total respondents (*n* = 246) chose “I don’t know”, in contrast to previous studies, where the most dominant adverse effect was allergic reaction [12,13]. Patients in the age range of “over 65” years were the ones who most marked this option.

As to knowledge of antibiotic resistance, the questions “Yes” or “Yes. I think it is an issue of global health importance” were chosen by 54 (22%) and 82 (33.3%), respectively, representing 136 (55.3%) of the total (*n* = 246), compared to previous studies, where 69.3% and 53.3%, respectively, said they were aware of the concept of antibiotic resistance [10,12].

#### 4.1.2. Group 2 and Group 3: Fifth-Year Dentistry Students and Professors

The antibiotic of choice for treatment, Amoxicillin, was the most chosen drug among professors and students and among the different specialties of the professors. However, there were differences in the choice of dosage and the combination with clavulanic acid. Amoxicillin 750 mg was the most predominant dosage, chosen by 64% of professors and 79.7% of students. It should be noted that 16% of professors chose the combination with clavulanic acid in some dosage compared to 5.3% of students. With regards to the specialty, professors with specialties in surgery or similar were the ones that most frequently chose amoxicillin 750 mg. In previous studies, students also chose amoxicillin as their first option, but in this case, the highest percentage was combined with clavulanic acid [9]. According to the European Society of Endodontics [11], the antibiotics of choice should be beta-lactams (penicillin V and amoxicillin). In the case of amoxicillin, 1 g of amoxicillin followed by 500 mg every 4–6 h, and in the hypothetical case that this therapy is ineffective, it should be replaced with penicillin V combined with metronidazole or amoxicillin with clavulanic acid. As previously mentioned with darobactin, future studies, in addition to focusing on the discovery or synthesis of new antibiotics, should support already active avenues of research such as chitosan, a multifactorial polymer of natural origin with antibacterial effects, mainly used in surgery and implantology, which could be used in other dental specialties if its current limitations continue to be improved [24]. It would also be interesting to strengthen the different avenues of research of existing drugs in different presentations, such as metronidazole, which has been shown to have antimicrobial capacity presented in an in situ forming matrix with camphor for the treatment of periodontitis [25].

For the prescription duration, most of the professors and students (80% and 91.1%), respectively, would prescribe the antibiotic for 7 days, with 3–5 days being the second option chosen by 20% of professors and 7.6% of students. In previous studies, students also chose 7 days in greater proportion, with 69%, where the remaining percentage, unlike our study, indicated that they would prescribe it for a longer time [9]. The current guideline for therapeutic duration recommends that antibiotics be discontinued when symptoms disappear and there is evidence of healing. Therefore, patients should be examined 2 or 3 days after the first administration to determine when treatment should be discontinued [11].

In patients with a penicillin allergy, comparing the choice of professors and students, clindamycin and azithromycin are the most commonly indicated, but there are differences with respect to the dosage. According to the specialty, professors with a specialty in surgery or similar and those with a specialty in periodontology preferred azithromycin 500 mg. Students in previous surveys had fewer differences between them when choosing a drug for patients allergic to penicillin, 99% selected clindamycin 300 mg [9]. If a true penicillin allergy is confirmed, current evidence recommends replacing amoxicillin with clarithromycin, azithromycin or clindamycin, with a dose of 500 mg followed by 250 mg of clarithromycin every 12 h, 500 mg followed by 250 mg of azithromycin once a day or 600 mg followed by 300 mg of clindamycin every 6 h [11]. However, other authors, such as Mahmoud et al. (2021), chose clindamycin as the antibiotic of choice [26]. Clindamycin has demonstrated good efficacy against aerobic cocci and most anaerobes, including penicillin-resistant species, with evidence showing that its efficacy in odontogenic infections is comparable to that of amoxicillin/clavulanic acid. However, it is important to note that clindamycin is a bacteriostatic agent with a narrow spectrum, primarily targeting anaerobic pathogens, whereas amoxicillin is a bactericidal agent with a broad spectrum of activity [27]. On the other hand, the Spanish Society of Implants (SEI) indicated that the use of clindamycin in implant placement has shown a significant risk of implant failure, associated with the failure of osseointegration and a risk of infection up to six times higher in patients who were administered amoxicillin [28].

Regarding clinical situations for antibiotics, both professors (91.1%) and students (92%), taking into account the specialties of the professors, were very clear that pulp necrosis with symptomatic apical periodontitis or abscess, with moderate or severe symptoms, requires antibiotic treatment, not being so with the other options, with more heterogeneous data. Other studies revealed the same result, chosen by 90.2%.

Amongst the answers to clinical situations where antibiotic prophylaxis would be indicated, the risk of bacterial endocarditis was the most prevalent both in professors, exceeding 80% in all, and in students (97.5%). For the rest of the situations, more heterogeneous responses were obtained. According to the current evidence, it should only be considered when a benefit has been demonstrated or when there is consensus for such use. These studies recommend making an individualized global medical evaluation and always, when in doubt, consulting a doctor [11].

One study revealed that dental students who graduated during COVID-19 felt they had a lower level of self-confidence in clinical skills than students in pre-pandemic graduating classes [29]. Another study related to COVID-19 conducted on the same university campus as our study revealed that out of 160 students of different health studies, including dentistry, 82% of students reported having stress, anxiety or distress [30]. This may also lead to a deficit in antibiotic prescription, which would be interesting to study in an alternative line of research.

The limitations of this study include the fact that it is a descriptive, cross-sectional study and that causality cannot be established; it simply describes a specific situation. On the other hand, the conclusions of this research, being a pilot study, may not be applicable to a wider population without first conducting additional studies that include larger and more diverse samples since the population studied is limited to both patients and health professionals at the Dental Hospital of the University of Barcelona. Although the study provides valuable information and may indicate important trends, its results cannot be extrapolated to the general population without taking additional precautions and encouraging complementary studies to validate the findings by means of a more representative sample.

## 5. Conclusions

Patients expect antibiotics to be prescribed in situations where they are not necessary, such as when there is dental pain, which generates distrust towards professionals when they are not prescribed.

Patients with lower educational levels are more likely to expect antibiotic prescriptions when they are not necessary, have less knowledge of the side effects of antibiotics, as well as the concept of antibiotic resistance, and tend to self-medicate more frequently. In addition, younger participants believed that antibiotic treatment is especially useful for pain treatment.

More time should be dedicated to thoroughly explaining to patients why antibiotics are or are not needed in each case, as well as implementing educational plans for the population. Additionally, the educational plan should be reviewed to improve the training of students in the use of antibiotics, as well as implementing continuous training throughout their professional lives. There are variations in the choice, dosage and days of the prescription of drugs both between dental specialty professors and students and among professors of different specialties. In clinical situations for the prescription of antibiotics, students and professors choose different prescription situations, with some discrepancies to current guidelines. 

## Figures and Tables

**Table 1:** Demographic data with the gender, age and educational level of Group 1: Patients.

Gender		Age		Educational Level	
Male Female Other Total	114 (46.3%) 131 (53.3%) 1 (0.4%) 246 (100%)	18–28 years 25–30 years 30–35 years 35–45 years 45–55 years 55–65 years 65–85 years Total	22 (8.9%) 13 (5.3%) 17 (6.9%) 34 (13.8%) 44 (17.9%) 51 (20.7%) 65 (26.9%) 246 (100%)	Without studies Primary or general basic education Compulsory secondary education or * BUP High School Secondary vocational training Higher vocational training Bachelor or university degree Postgraduate or university diploma Doctoral Thesis Total	3 (1.2%) 58 (23.6%) 38 (15.4%) 40 (16.3%) 38 (15.4%) 30 (12.2%) 30 (12.2%) 5 (2%) 4 (1.6%) 246 (100%)

* BUP: “Unified and Polyvalent Baccalaureate”.

**Table 2:** Demographic data with the gender, age and educational level of Group 2 and Group 3: Students and Professors.

Group 2: Students			
Gender (%)		Age (%)	
Male Female Total	18 (22.80%) 61 (77.20%) 79 (100%)	20–25 25–30 30–35 35–45 45–55 Total	53 (67.10%) 20 (25.30%) 3 (4%) 2 (2.5%) 1 (1.30%) 79 (100%)
**Group 3: Professors**			
**Gender (%)**		**Age (%)**	
Male Female Total	23 (46%) 27 (54%) 50 (100%)	25–30 30–35 35–45 45–55 55–70 Total	2 (4%) 12 (24%) 10 (20%) 12 (24%) 14 (28%) 50 (100%)

## Data Availability

The original contributions presented in this study are included in the article/Supplementary Material. Further inquiries can be directed to the corresponding authors.

## Supplementary Materials

The following supporting information can be downloaded at: https://www.mdpi.com/article/10.3390/jcm14072179/s1, Table S1. Contingency table. Group 1 (Patients); Table S2. Contingency table. Group 1 (Patients); Table S3. Contingency table. Group 1 (Patients); Table S4. Contingency table. Group 2–3 (Students and professors); Table S5. Contingency table. Group 3 (Professors); Table S6. Contingency table. Group 3 (Professors); Table S7. Contingency table. Group 2–3 (Students-Professors); Table S8. Contingency table. Group 2–3 (Students-Professors).
