# Supplementary material for: Knowledge of Antibiotic Management in Surgery, Periodontics and Endodontics Among Patients, Students and Dentistry Professors: A Cross-Sectional Study at the University of Barcelona (Spain)
Source: J Clin Med. 2025 Mar 22;14(7):2179. doi: 10.3390/jcm14072179 (PMC11989375; doi:10.3390/jcm14072179)
Supplement: Supplementary file 1 [file jcm-14-02179-s001.zip › jcm-3507024-supplementary.pdf]

Table S1. Contingency table. Group 1 (Patients)

GENDER

Question	Variable	Male	Female	Total (%)	P-value
	Variable	Frequency and percentage			*P-value*
4.-Have you ever had a root canal? (removing, extracting, or killing the nerve).	Yes	77 (67,5 %)	97 (74 %)	174 (71 %)	0,428
	No	36 (31,6 %)	32 (24,4 %)	68 (27,8 %)	
	I don't remember	1 (0,9 %)	2 (1,5 %)	3 (1,2 %)	
	Total	114 (100 %)	131 (100 %)	245 (100 %)	
	Variable	Frequency and percentage			*P-value*
5.-Beforeperforming the root canal treatment, do you think it is necessary to take antibiotics?	Yes	38 (33,3 %)	48 (36,6 %)	86 (35,1 %)	0,588
	No	76 (66,7 %)	83 (63,4 %)	159 (64,9 %)	
	Total	114 (100 %)	131 (100 %)	245 (100 %)	
	Variable	Frequency and percentage			*P-value*
6.-After completing the root canal treatment, do you think it is necessary to take antibiotics?	Yes	44 (38,6 %)	59 (45 %)	103 (42 %)	0,308
	No	70 (61,4 %)	72 (55 %)	142 (58 %)	
	Total	114 (100 %)	131 (100 %)	245 (100 %)	
	Variable	Frequency and percentage			*P-Value*
7.-If the professional does not prescribe antibiotics before or after, would you ask him why he does not prescribe them?	Yes	45 (39,5 %)	48 (36,6 %)	93 (38 %)	0,649
	No	69 (60,5 %)	83 (63,4 %)	152 (62 %)	
	Total	114 (100 %)	131 (100 %)	245 (100 %)	

Question	Variable	Male	Female	Total (%)	Total
	Variable	Frequency and percentage			*P-value*
15.-What side effects do you think you may experience if you use antibiotics? You can choose more than one option	Nausea or vomiting	21 (18,4 %)	27 (20,6 %)	48 (19,6 %)	0,667
	Diarrhea	24 (21,1 %)	43 (32,8 %)	67 (27,3 %)	**0,039**
	Fever	8 (7 %)	14 (10,7 %)	22 (9 %)	0,316
	Fungal infections	13 (11,4 %)	34 (26 %)	47 (19,2 %)	**0,004**
	Allergic reaction	29 (25,4 %)	31 (23,7 %)	60 (24,5 %)	0,747
	None of the above	15 (13,2 %)	20 (15,3 %)	35 (14,3 %)	0,638
	I don't know	52 (45,6 %)	40 (30,5 %)	92 (37,6 %)	**0,015**
	Total	114 (100 %)	131 (100 %)	245 (100 %)	
	Variable	Frequency and percentage			*P-value*
16.-When you take antibiotics, for how long do you take them?	1 day	0 (0 %)	1 (0,8 %)	1 (0,4 %)	0,195
	2 days	4 (3,5 %)	2 (1,5 %)	6 (2,5 %)	
	3-5 days	31 (27,4 %)	24 (18,3 %)	55 (22,5 %)	
	7 days	68 (60,2 %)	86 (65,6 %)	153 (62, 7 %)	
	2 weeks or more	0 (0 %)	3 (2,3 %)	3 (1,2 %)	
	Other	10 (8,8 %)	15 (11,5 %)	25 (10,2 %)	
	Total	113 (100 %)	131 (100 %)	244 (100 %)	
	Variable	Frequency and percentage			*P-value*

	Variable	Frequency and percentage			*P-value*
8.-If your dentist tells you that you have a dental infection, do you expect him to prescribe antibiotics?	Yes	104 (91,2 %)	120 (91,6 %)	224 (91,4 %)	0,917
	No	10 (8,8 %)	11 (8,4 %)	21 (8,6 %)	
	Total	114 (100 %)	131 (100 %)	245 (100 %)	

	Variable	Frequency and percentage			*P-value*
9.-If the professional does not prescribe antibiotics, would you look for another practitioner and ask, or not, why your doctor did not prescribe antibiotics??	Yes, I would look for another dentist and not ask why my doctor didn't prescribe antibiotics.	11 (9,6 %)	8 (6,1 %)	19 (7,8 %)	0,585
	Yes, I would look for another dentist and also ask why my doctor has not prescribed antibiotics.	24 (21,1 %)	29 (22,1 %)	53 (21,6 %)	
	No	79 (69,3 %)	94 (71,8 %)	173 (70,6 %)	
	Total	114 (100 %)	131 (100 %)	245 (100 %)	

	Variable	Frequency and percentage			*P-value*
10.-If you suffer from dental pain, do you expect your dentist to prescribe antibiotics?	Yes	52 (45,6 %)	41 (31,3 %)	93 (38 %)	**0,021**
	No	62 (54,4 %)	90 (68,7 %)	152 (62 %)	
	Total	114 (100 %)	131 (100 %)	245 (100 %)	

	Variable	Frequency and percentage			*P-value*
11.-If you suffer from a dental infection, do you expect your dentist to prescribe antibiotics?	Yes	102 (89,5 %)	120 (91,6 %)	222 (90,6 %)	0,569
	Yes	12 (10,5 %)	11 (8,4 %)	23 (9,4 %)	
	Total	114 (100 %)	131 (100 %)	245 (100 %)	

	Variable	Frequency and percentage			*P-value*
	Yes	34	32	66	

17.-You have knowledge of antibiotic resistance	Yes	23 (20,2 %)	31 (23,8 %)	54 (22,1 %)	0,771
	Yes. I think it is an issue of global health importance	40 (35,1 %)	42 (32,3 %)	82 (33,6%)	
	No	51 (44,7 %)	57 (43,8 %)	108 (44,3 %)	
	Total	114 (100 %)	131 (100 %)	245 (100 %)	

	Variable	Frequency and percentage			*P-value*
18.-Have you ever had any teeth extracted?	Yes	98 (86 %)	113 (86,3 %)	211 (86,1 %)	0,947
	No	16 (14 %)	18 (13,7 %)	34 (13,9 %)	
	Total	114 (100 %)	131 (100 %)	245 (100 %)	

	Variable	Frequency and percentage			*P-value*
19.-Have you ever had any more complicated oral surgery?	Extraction of traumatic tooth (fractured tooth, tooth associated with a cyst, enclosed tooth)	25 (21,9 %)	25 (19,1 %)	50 (20,4 %)	0,581
	Sutura	19 (16,7 %)	12 (9,2 %)	31 (12,7 %)	0,078
	Bone regeneration	4 (3,5 %)	5 (3,8 %)	9 (3,7 %)	0,898
	Others	10 (8,8 %)	12 (9,2 %)	22 (9 %)	0,916
	No	69 (60,5 %)	80 (61,1 %)	149 (60,8 %)	0,931

	Variable	Frequency and percentage			*P-value*
20.-Before extracting the tooth, do you think it is necessary to take antibiotics?	Yes	17 (14,9 %)	14 (10,7 %)	31 (12,7 %)	0,708
	No	47 (41,2 %)	53 (40,5 %)	100 (40,8 %)	
	On some occasions	40 (35,1 %)	49 (37,4 %)	89 (36,3 %)	
	I do not know	10	10	25	

12.-Have you ever self-medicated with antibiotics for toothache?		(29,8 %)	(24,4 %)	(26,9 %)	0,343
	No	80 (70,2 %)	99 (75,6 %)	178 (73,1 %)	
	Total	114 (100 %)	131 (100 %)	245 (100 %)	

	Variable	Frequency and percentage			*P-value*
13.-Have you ever self-medicated with an antibiotic for a dental infection?	Yes	34 (29,8 %)	38 (29 %)	72 (29,4 %)	0,552
	No	80 (70,2 %)	93 (71 %)	173 (70,6 %)	
	Total	114 (100 %)	131 (100 %)	245 (100 %)	

	Variable	Frequency and percentage			*P-value*
14.-What benefits do you think there are to taking antibiotics? You can choose more than one option	Reduces pain	43 (37,7 %)	50 (38,2 %)	93 (38 %)	0,942
	Reduces inflammation	46 (40,4 %)	57 (43,5 %)	103 (42 %)	0,617
	Reduces chance of infection	73 (64 %)	78 (59,5 %)	151 (61,6 %)	0,471
	Offers no benefit	1 (0,9 %)	3 (2,3 %)	4 (1,6 %)	0,384
	Improves oral health	14 (12,3 %)	11 (8,4 %)	25 (10,2 %)	0,316
	I don't know	11 (9,7 %)	12 (9,1 %)	23 (9,4 %)	0,745
	Total	114 (100 %)	131 (100 %)	245 (100 %)	

		(8,8 %)	(11,5 %)	(10,2 %)	
	Total	114 (100 %)	131 (100 %)	245 (100 %)	

	Variable	Frequency and percentage			*P-value*
21.-After extracting the tooth, do you think it is necessary to take antibiotics?	Yes	24 (21,1 %)	26 (19,8 %)	50 (20,4 %)	0,492
	No	25 (21,9 %)	31 (23,7 %)	56 (22,9 %)	
	On some occasions	52 (45,6 %)	66 (50,4 %)	118 (48,2 %)	
	I do not know	13 (11,4 %)	8 (6,1 %)	21 (8,6 %)	
	Total	114 (100 %)	131 (100 %)	245 (100 %)	

	Variable	Frequency and percentage			*P-value*
22.-Do you think that antibiotics would be indicated for patients with diseases that are more likely to cause complications after tooth extrations?	Yes	59 (51,8 %)	43 (32,8 %)	102 (41,6 %)	**0,007**
	No	16 (14 %)	19 (14,5 %)	35 (14,3 %)	
	On some occasions	39 (34,2 %)	69 (52,7 %)	108 (44,1 %)	
	Total	114 (100 %)	131 (100 %)	245 (100 %)	

	Variable	Frequency and percentage			*P-value*
23.-Have you ever self-medicated with antibiotics before or after having a tooth removed?	Yes	16 (14 %)	22 (16,8 %)	38 (15,5 %)	0,552
	No	98 (86 %)	109 (83,2 %)	207 (84,5 %)	
	Total	114 (100 %)	131 (100 %)	245 (100 %)	

	Variable	Frequency and percentage			*P-value*
24.-Have you ever had any implant surgery that you consider simple?	Yes	9 (7,9 %)	18 (13,7 %)	27 (11 %)	0,145
	No	105 (92,1 %)	113 (86,3 %)	218 (89 %)	
	Total	114 (100 %)	131 (100 %)	245 (100 %)	

	Variable	Frequency and percentage			*P-value*

							25.-Have you had implant surgery that you consider complex?	Yes. Orthopedic rehabilitation-related surgery	1 (0,9 %)	0 (0 %)	1 (0,4 %)	0,283
								Yes, surgery related to bone regeneration.	0 (0 %)	2 (1,5 %)	2 (0,8 %)	0,185
								Yes. Implant-related surgery	2 (1,8 %)	2 (1,5 %)	4 (1,6 %)	0,888
								Yes. Surgery related to molar exodontics	0 (0 %)	1 (0,8 %)	1 (0,4 %)	0,350
								Yes. Surgery related to failed implants	0 (0 %)	1 (0,8 %)	1 (0,4 %)	0,350
								No	109 (95,6 %)	125 (95,4 %)	234 (95,5 %)	0,942
								Total	114 (100 %)	131 (100 %)	245 (100 %)	

Table S2. Contingency table. Group 1 (Patients)							
AGE							
	18 - 35	35 - 45	45 - 55	55 - 65	> 65	Total (%)	
Variable	Frequency and percentage						*P-value*
4. Have you ever had a root canal? (removing, extracting, or killing the nerve).							
Yes	24 (46,2 %)	22 (64,7 %)	37 (84,1 %)	39 (76,5 %)	52 (80 %)	174 (70,7 %)	**0,002**
No	27 (51,9 %)	11 (32,4%)	7 (15,9 %)	12 (23,5 %)	12 (18,5 %)	69 (28 %)	
I don't remember	1 (1,9 %)	1 (2,9 %)	0 (0 %)	0 (0 %)	1 (1,5 %)	3 (1,2 %)	
Total	52 (100 %)	34 (100 %)	44 (100 %	51 (100 %)	65 (100 %)	246 (100 %)	
5. Before performing the root canal treatment, do you think it is necessary to take antibiotics?							
Variable	Frequency and percentage						*P-value*
Yes	17 (32,7 %)	8 (23,5%)	21 (47,7 %)	15 (29,4 %)	25 (38,5 %)	86 (35 %)	0,180
No	35 (67,3 %)	26 (76,5 %)	23 (52,3 %)	36 (70,6 %)	40 (61,50 %)	160 (65 %)	
Total	52 (100 %)	34 (100 %)	44 (100 %	51 (100 %)	65 (100 %)	246 (100 %)	
6. After completing the root canal treatment, do you think it is necessary to take antibiotics?							
Variable	Frequency and percentage						*P-value*
Yes	28 (53,8 %)	11 (32,4 %)	22 (50 %)	20 (39,2 %)	22 (33,8 %)	103 (41,9 %)	0,117
No	24 (46,2 %)	23 (67,6 %)	22 (50 %)	31 (60,8 %)	43 (66,2 %)	143 (58,1 %)	
Total	52 (100 %)	34 (100 %)	44 (100 %	51 (100 %)	65 (100 %)	246 (100 %)	
7. If the professional does not prescribe antibiotics before or after, would you ask him why he does not prescribe them?							

Variable	Frequency and percentage						*P-value*
Yes	15 (28,8 %)	11 (32,4 %)	22 (50 %)	24 (47,1 %)	21 (32,3 %)	93 (37,8 %)	0,104
No	37 (71,2 %)	23 (67,6 %)	22 (50 %)	27 (52,9 %)	44 (67,7 %)	153 (62,2 %)	
Total	52 (100 %)	34 (100 %)	44 (100 %	51 (100 %)	65 (100 %)	246 (100 %)	

8. If your dentist tells you that you have a dental infection, do you expect him to prescribe antibiotics?

Variable	Frequency and percentage						*P-value*
Yes	45 (86,5 %)	31 (91,2 %)	41 (93,2 %)	48 (94,1 %)	59 (90,8 %)	224 (91,1 %)	0,710
No	7 (13,5 %)	3 (8,8 %)	3 (6,8 %)	3 (5,9 %)	6 (9,2 %)	22 (8,9 %)	
Total	52 (100 %)	34 (100 %)	44 (100 %	51 (100 %)	65 (100 %)	246 (100 %)	

9. If the professional does not prescribe antibiotics, would you look for another practitioner and ask, or not, why your doctor did not prescribe antibiotics??

Variable	Frequency and percentage						*P-value*
Yes, I would look for another dentist and not ask why my doctor didn't prescribe antibiotics.	1 (1,9 %)	6 (17,6 %)	1 (2,3%)	5 (9,8 %)	6 (9,2 %)	19 (7,7 %)	0,272
Yes, I would look for another dentist and also ask why my doctor has not prescribed antibiotics.	12 (23,1 %)	6 (17,6 %)	9 (20,5%)	12 (23,5 %)	14 (21,5 %)	53 (21,5 %)	
No	39 (75 %)	22 (64,7 %)	34 (77,3 %)	34 (66,7 %)	45 (69,2 %)	174 (70,7 %)	
Total	52 (100 %)	34 (100 %)	44 (100 %	51 (100 %)	65 (100 %)	246 (100 %)	

10. If you suffer from dental pain, do you expect your dentist to prescribe antibiotics?

Variable	Frequency and percentage						*P-value*
Yes	19 (36,5 %)	11 (32,4 %)	13 (29,5 %)	24 (47,1 %)	26 (40 %)	93 (37,8 %)	0,443
No	33 (63,5 %)	23 (67,6 %)	31 (70,5 %)	27 (52,9 %)	39 (60 %)	153 (62,2 %)	
Total	52 (100 %)	34 (100 %)	44 (100 %	51 (100 %)	65 (100 %)	246 (100 %)	

11. If you suffer from a dental infection, do you expect your dentist to prescribe antibiotics?							
Variable	Frequency and percentage						*P-value*
Yes	48 (92,3 %)	30 (88,2 %)	38 (86,4 %)	46 (90,2 %)	60 (92,3 %)	222 (90,2 %)	0,831
No	4 (7,7 %)	4 (11,8 %)	6 (13,6 %)	5 (9,8 %)	5 (7,7 %)	24 (9,8 %)	
Total	52 (100 %)	34 (100 %)	44 (100 %	51 (100 %)	65 (100 %)	246 (100 %)	

12. Have you ever self-medicated with antibiotics for toothache?							
Variable	Frequency and percentage						*P-value*
Yes	15 (28,8%)	9 (26,5 %)	16 (36,4 %)	13 (25,5 %)	13 (20 %)	66 (26,8 %)	0,375
No	37 (71,2 %)	25 (73,5 %)	28 (63,7 %)	38 (74,5 %)	52 (80 %)	180 (73,2 %)	
Total	52 (100 %)	34 (100 %)	44 (100 %	51 (100 %)	65 (100 %)	246 (100 %)	

13. Have you ever self-medicated with an antibiotic for a dental infection?							
Variable	Frequency and percentage						*P-value*
Yes	11 (21,2 %)	11 (32,4 %)	19 (43,2 %)	12 (23,5 %)	19 (29,2 %)	72 (29,3 %)	0,162
No	41 (78,8 %)	23 (67,6 %)	25 (56,8 %)	39 (76,5 %)	46 (70,8 %)	173 (70,7 %)	
Total	52 (100 %)	34 (100 %)	44 (100 %	51 (100 %)	65 (100 %)	246 (100 %)	

14. What benefits do you think there are to taking antibiotics? You can choose more than one option							
Variable	Frequency and percentage						*P-value*
Reduces pain	30 (57,7 %)	11 (32,4 %)	18 (40,9 %)	20 (39,2 %)	14 (21,5 %)	93 (37,8 %)	**0,002**
Reduces inflammation	31 (59,6 %)	9 (26,5 %)	20 (45,5 %)	23 (45,1 %)	20 (30,8 %)	103 (41,9 %)	**0,008**
Reduces chance of infection	30 (57,7 %)	18 (52,9 %)	30 (68,2 %)	33 (64,7 %)	40 (61,5 %)	151 (61,4 %)	0,66
Offers no benefit	1 (1,9 %)	0 (0 %)	1 (2,3 %)	0 (0 %)	2 (3,1 %)	4 (1,6 %)	0,662
Improves oral health	5 (9,6 %)	0 (0 %)	5 (11,4 %)	7 (13,7 %)	8 (12,3 %)	25 (10,2 %)	0,290

I don't know	5 (9,6 %)	2 (5,9 %)	3 (6,8 %)	4 (7,8 %)	10 (12,3 %)	24 (9,7 %)	0,662
Total	52 (100 %)	34 (100 %)	44 (100 %	51 (100 %)	65 (100 %)	246 (100 %)	

15. What side effects do you think you may experience if you use antibiotics? You can choose more than one option

Variable	Frequency and percentage						*P-value*
Nausea or vomiting	17 (32,7 %)	7 (20,6 %)	9 (20,5 %)	12 (23,5 %)	4 (6,2 %)	49 (19,9 %)	**0,009**
Diarrhea	23 (44,2 %)	8 (23,5 %)	17 (38,6 %)	12 (23,5 %)	9 (12,3 %)	68 (27,6 %)	**0,001**
Fever	11 (21,2 %)	1 (2,9 %)	2 (4,5 %)	4 (7,8 %)	4 (6,2 %)	22 (8,9 %)	0,012
Fungal infections	17 (32,7 %)	7 (20,6 %)	10 (22,7 %)	8 (15,7 %)	6 (9,2 %)	48 (19,5 %)	**0,028**
Allergic reaction	20 (38,5 %)	8 (23,5 %)	10 (22,7 %)	14 (27,5 %)	8 (12,3 %)	60 (24,4 %)	**0,026**
None of the above	1 (1,9 %)	5 (14,7 %)	6 (13,6 %)	7 (13,7 %)	16 (24,6 %)	35 (14,2 %)	**0,016**
I don't know	16 (30,8 %)	13 (38,2 %)	12 (27,3 %)	19 (37,3 %)	32 (49,2 %)	92 (37,4 %)	0,147
Total	52 (100 %)	34 (100 %)	44 (100 %	51 (100 %)	65 (100 %)	246 (100 %)	

16. When you take antibiotics, for how long do you take them

Variable	Frequency and percentage						*P-value*
1 day	1 (2 %)	0 (0 %)	0 (0 %)	0 (0 %)	0 (0 %)	1 (0,4 %)	0,496
2 days	2 (3,9 %)	0 (0 %)	0 (0 %)	3 (5,9 %)	1 (1,5 %)	6 (2,4 %)	
3-5 days	13 (25,5 %)	13 (38,2 %)	6 (38,2 %)	8 (15,7 %)	15 (23,1 %)	55 (22,4 %)	
7 days	29 (56,9 %)	17 (50 %)	32 (72,7 %)	33 (64,7 %)	44 (67,7 %)	155 (63,3 %)	
2 weeks or more	1 (2 %)	0 (0 %)	0 (0 %)	1 (2 %)	1 (1,5 %)	3 (1,2 %)	
Other	5 (9,8 %)	4 (11,8 %)	6 (13,6 %)	6 (11,8 %)	4 (6,2 %)	25 (10,2 %)	
Total	51 (100 %)	34 (100 %)	44 (100 %	51 (100 %)	65 (100 %)	245 (100 %)	

17. You have knowledge of antibiotic resistance							
Variable	Frequency and percentage						*P-value*
Yes	7 (13,5 %)	7 (21,2 %)	10 (22,7 %)	11 (21,6 %)	19 (29,2 %)	54 (22 %)	0,530
Yes. I think it is an issue of global health importance	22 (42,3 %)	11 (33,3 %)	16 (36,4 %)	18 (35,3 %)	15 (23,1 %)	82 (33,5 %)	
No	23 (44,2 %)	15 (45,5 %)	18 (40,9 %)	22 (43,1 %)	31 (47,7 %)	109 (44,5 %)	
Total	52 (100 %)	34 (100 %)	44 (100 %	51 (100 %)	65 (100 %)	245 (100 %)	

18. Have you ever had any teeth extracted?							
Variable	Frequency and percentage						*P-value*
Yes	35 (67,3 %)	29 (85,3 %)	39 (88,6 %)	46 (90,2 %)	63 (96,9 %)	212 (86,2 %)	0.00
No	17 (32,7 %)	5 (14,7 %)	5 (11,4 %)	5 (9,8 %)	2 (3,1 %)	34 (13,8 %)	
Total	52 (100 %)	34 (100 %)	44 (100 %	51 (100 %)	65 (100 %)	246 (100 %)	

19. Have you ever had any more complicated oral surgery?							
Variable	Frequency and percentage						*P-value*
Extraction of traumatic tooth (fractured tooth, tooth associated with a cyst, enclosed tooth)	5 (9,6 %)	8 (23,5 %)	13 (29,5 %)	12 (23,5 %)	12 (18,5 %)	50 (20,3 %)	0,154
Sutura	2 (3,8 %)	3 (8,8 %)	4 (9,1 %)	9 (17,6 %)	13 (20 %)	31 (12,6 %)	0,062
Bone regeneration	1 (1,9 %)	1 (2,9 %)	2 (4,5 %)	1 (2 %)	4 (6,2 %)	9 (3,7 %)	0,707
Others	6 (11,5 %)	3 (8,8 %)	2 (4,5 %)	5 (9,8 %)	6 (9,2 %)	22 (8,9 %)	0,822
No	39 (75 %)	21 (61,8 %)	27 (61,4 %)	28 (54,9 %)	35 (53,8 %)	150 (61 %)	0,165
Total	52 (100 %)	34 (100 %)	44 (100 %	51 (100 %)	65 (100 %)	246 (100 %)	

20. Before extracting the tooth, do you think it is necessary to take antibiotics?							
Variable	Frequency and percentage						*P-value*
Yes	8 (15,4 %)	1 (2,9 %)	8 (18,2 %)	8 (15,7 %)	6 (9,2 %)	31 (12,6 %)	0,476

No	18 (34,6 %)	18 (52,9 %)	19 (43,2 %)	20 (39,2 %)	26 (40 %)	101 (41,1 %)	
On some occasions	20 (38,5 %)	9 (26,5 %)	15 (34,1 %)	19 (37,3 %)	26 (40 %)	89 (36,2 %)	
I don't know	6 (11,5 %)	6 (17,6 %)	2 (4,5 %)	4 (7,8 %)	7 (10,8 %)	25 (10,2 %)	
Total	52 (100 %)	34 (100 %)	44 (100 %	51 (100 %)	65 (100 %)	246 (100 %)	

21. After extracting the tooth, do you think it is necessary to take antibiotics?

Variable	Frequency and percentage						*P-value*
Yes	16 (30,8 %)	8 (23,5 %)	9 (20,5 %)	10 (19,6 %)	7 (10,8 %)	50 (20,3 %)	0,518
No	9 (17,3 %)	9 (26,5 %)	10 (22,7 %)	11 (21,6 %)	18 (27,7 %)	57 (23,2 %)	
On some occasions	22 (42,3 %)	13 (38,2 %)	23 (52,3 %)	27 (52,9 %)	33 (50,8 %)	118 (48 %)	
I don't know	5 (9,6 %)	4 (11,8 %)	2 (4,5 %)	3 (5,9 %)	7 (10,8 %)	21 (8,5 %)	
Total	52 (100 %)	34 (100 %)	44 (100 %)	51 (100 %)	65 (100 %)	246 (100 %)	

22. Do you think that antibiotics would be indicated for patients with diseases that are more likely to cause complications after tooth extrations?

Variable	Frequency and percentage						*P-value*
Yes	18 (34,6 %)	16 (47,1 %)	16 (36,4 %)	24 (47,1 %)	28 (43,1 %)	102 (41,5 %)	0,164
No	4 (7,7 %)	4 (11,8 %)	9 (20,5 %)	4 (7,8 %)	14 (21,5 %)	35 (14,2 %)	
On some occasions	30 (57,7 %)	14 (41,2 %)	19 (43,2 %)	23 (45,1 %)	23 (35,4 %)	109 (44,3 %)	
Total	52 (100 %)	34 (100 %)	44 (100 %	51 (100 %)	65 (100 %)	246 (100 %)	

23. Have you ever self-medicated with antibiotics before or after having a tooth removed?

Variable	Frequency and percentage						*P-value*
Yes	6 (11,5 %)	3 (8,8 %)	9 (20,5 %)	5 (9,8 %)	15 (23,1 %)	38 (15,4 %)	0,151
No	46 (88,5 %)	31 (91,2 %)	35 (79,5 %)	46 (90,2 %)	50 (76,9 %)	208 (84,6 %)	

Total	52 (100 %)	34 (100 %)	44 (100 %	51 (100 %)	65 (100 %)	246 (100 %)	

24. Have you ever had any implant surgery that you consider simple?

Variable	Frequency and percentage						P-value
Yes	2 (3,8 %)	3 (8,8 %)	8 (18,2 %)	4 (7,8 %)	10 (15,4 %)	27 (11 %)	0,135
No	50 (96,2 %)	31 (91,2 %)	36 (81,8 %)	47 (92,2 %)	55 (84,6 %)	219 (89 %)	
Total	52 (100 %)	34 (100 %)	44 (100 %	51 (100 %)	65 (100 %)	246 (100 %)	

25. Have you had implant surgery that you consider complex?

Variable	Frequency and percentage						*P-value*
Yes. Orthopedic rehabilitation-related surgery	0 (0 %)	0 (0 %)	0 (0 %)	0 (0 %)	1 (1,5 %)	1 (0,4 %)	0,593
Yes, surgery related to bone regeneration.	1 (1,9 %)	0 (0 %)	0 (0 %)	0 (0 %)	1 (1,5 %)	2 (0,8 %)	0,685
Yes. Implant-related surgery	0 (0 %)	1 (2,9 %)	1 (2,3 %)	0 (0 %)	2 (3,1 %)	4 (1,6 %)	0,551
Yes. Surgery related to molar exodontics	0 (0 %)	0 (0 %)	1 (2,3 %)	0 (0 %)	0 (0 %)	1 (0,4 %)	0,330
Yes. Surgery related to failed implants	0 (0 %)	0 (0 %)	1 (2,3 %)	0 (0 %)	0 (0 %)	1 (0,4 %)	0,330
No	51 (98,1 %)	33 (97,1 %)	41 (93,2 %)	50 (98 %)	60 (92,3 %)	235 (95,5 %)	0,423
Total	52 (100 %)	34 (100 %)	44 (100 %	51 (100 %)	65 (100 %)	246 (100 %)	

Table S3. Contingency table. Group 1 (Patients)							
**EDUCATIONAL LEVEL**							
	**Without studies**	**Basic studies**	**Secondary studies**	**Vocational training**	**University or higher**	**Total**	**P-value**
4. Have you ever had a root canal? (removing, extracting, or killing the nerve).							
Variable	Frequency and percentage						*P-value*
Yes	1 (33,3 %)	43 (74,1 %)	55 (70,5 %)	53 (77,9 %)	22 (56,4 %)	174 (70,7 %)	0,337
No	2 (66,7 %)	15 (25,9 %)	22 (28,2 %)	14 (20,6 %)	16 (41 %)	69 (28 %)	
I don't remember	0 (0 %)	0 (0 %)	1 (1,3 %)	1 (1,5 %)	1 (2,6 %)	3 (1,2 %)	
Total	3 (100 %)	58 (100 %	78 (100 %)	68 (100 %)	39 (100 %)	246 (100 %)	
5. Before performing the root canal treatment, do you think it is necessary to take antibiotics?							
Variable	Frequency and percentage						*P-value*
Yes	2 (66,7 %)	23 (39,7 %)	26 (33,3 %)	23 (33,8 %)	12 (30,8 %)	86 (35 %)	0,677
No	1 (33,3 %)	35 (60,3 %)	52 (66,7 %)	45 (66,2 %)	27 (69,2 %)	160 (65 %)	
Total	3 (100 %)	58 (100 %	78 (100 %	68 (100 %)	39 (100 %)	246 (100 %)	
6. After completing the root canal treatment, do you think it is necessary to take antibiotics?							
Variable	Frequency and percentage						*P-value*
Yes	2 (66,7 %)	14 (24,1 %)	38 (48,7 %)	30 (44,1 %)	19 (48,7 %)	103 (41,9 %)	**0,031**
No	1 (33,3 %)	44 (75,9 %)	40 (51,3 %)	38 (55,9 %)	20 (51,3 %)	143 (58,1 %)	
Total	3 (100 %)	58 (100 %	78 (100 %	68 (100 %	39 (100 %)	246 (100 %)	

7. If the professional does not prescribe antibiotics before or after, would you ask him why he does not prescribe them?							
Variable	Frequency and percentage						*P-value*
Yes	2 (66,7 %)	20 (34,5 %)	29 (37,2 %)	28 (41,2 %)	14 (35,9 %)	93 (37,8 %)	0,784
No	1 (33,3 %)	38 (65,5 %)	49 (62,8 %)	40 (58,8 %)	25 (64,1 %)	153 (62,2 %)	
Total	3 (100 %)	58 (100 %)	78 (100 %)	68 (100 %)	39 (100 %)	246 (100 %)	

8. If your dentist tells you that you have a dental infection, do you expect him to prescribe antibiotics?							
Variable	Frequency and percentage						*P-value*
Yes	2 (66,7 %)	54 (93,1 %)	70 (89,7 %)	63 (92,6 %)	35 (89,7 %)	224 (91,1 %)	0,566
No	1 (33,3 %)	4 (6,9 %)	8 (10,3 %)	5 (7,4 %)	4 (10,3 %)	22 (8,9 %)	
Total	3 (100 %)	58 (100 %	78 (100 %	68 (100 %)	39 (100 %)	246 (100 %)	

9. If the professional does not prescribe antibiotics, would you look for another practitioner and ask, or not, why your doctor did not prescribe antibiotics??							
Variable	Frequency and percentage						*P-value*
Yes, I would look for another dentist and not ask why my doctor didn't prescribe antibiotics.	0 (0 %)	6 (10,3 %)	8 (10,3 %)	3 (4,4 %)	2 (5,1 %)	19 (7,7 %)	0,460
Yes, I would look for another dentist and also ask why my doctor has not prescribed antibiotics	1 (33,3 %)	8 (13,8 %)	20 (25,6 %)	18 (26,5 %)	6 (15,4 %)	53 (21,5 %)	
No	2 (66,7 %)	44 (75,9 %)	50 (64,1 %)	47 (69,1 %)	31 (79,5 %)	174 (70,7%)	
Total	3 (100 %)	58 (100 %)	78 (100 %)	68 (100 %)	39 (100 %)	246 (100 %)	

10. If you suffer from dental pain, do you expect your dentist to prescribe antibiotics?							
Variable	Frequency and percentage						*P-value*
Yes	3 (100 %)	28 (48,3 %)	32 (41 %)	22 (32, 4 %)	8 (20,5 %)	93 (37,8 %)	**0,008**
No	0 (0 %)	30 (51,7 %)	46 (59 %)	46 (67,6 %)	31 (79,5 %)	153 (62,2 %)	
Total	3 (100 %)	58 (100 %)	78 (100 %)	68 (100 %)	39 (100 %)	246 (100 %)	

11. If you suffer from a dental infection, do you expect your dentist to prescribe antibiotics?							
Variable	Frequency and percentage						*P-value*
Yes	3 (100 %)	55 (94,8 %)	69 (88,5 %)	61 (89,7 %)	34 (87,2 %)	222 (90,2 %)	0,658
No	0 (0 %)	3 (5,2 %)	9 (11,5 %)	7 (10,3 %)	5 (12,8 %)	24 (9,8 %)	
Total	3 (100 %)	58 (100 %	78 (100 %)	68 (100 %)	39 (100 %)	246 (100 %)	

12. Have you ever self-medicated with antibiotics for toothache?							
Variable	Frequency and percentage						*P-value*
Yes	2 (66,7 %)	16 (27,6 %)	24 (30,8 %)	12 (17,6 %)	12 (30,8 %)	66 (26,8 %)	0,296
No	1 (33,3 %)	42 (72,4 %)	54 (69,2 %)	56 (82,4 %)	27 (69,2 %)	180 (73,2%)	
Total	3 (100 %)	58 (100 %	78 (100 %)	68 (100 %)	39 (100 %)	246 (100 %)	

13. Have you ever self-medicated with an antibiotic for a dental infection?							
Variable	Frequency and percentage						*P-value*
Yes	1 (33,3 %)	20 (34,5 %)	27 (34,6 %)	15 (22,1 %)	9 (23,1 %)	72 (29,3 %)	0,462
No	2 (66,7 %)	38 (65,5 %)	51 (65,4 %)	53 (77,9 %)	30 (76,9 %)	174 (70,7 %)	
Total	3 (100 %)	58 (100 %	78 (100 %)	68 (100 %)	39 (100 %)	246 (100 %)	

14. What benefits do you think there are to taking antibiotics? You can choose more than one option							
Variable	Frequency and percentage						*P-value*
Reduces pain	1 (33,3 %)	21 (36,2 %)	30 (38,5 %)	28 (41,2 %)	13 (33,3 %)	93 (37,8 %)	0,943
Reduces inflammation	1 (33,3 %)	26 (44,8 %)	32 (41 %)	30 (44,1 %)	14 (35,9 %)	103 (41,9 %)	0,905
Reduces chance of infection	1 (33,3 %)	35 (60,3 %)	51 (65,4 %)	39 (57,4 %)	25 (64,1 %)	151 (61,4 %)	0,711
Offers no benefit	0 (0 %)	2 (3,4 %)	0 (0 %)	1 (1,5 %)	1 (2,6 %)	4 (1,6 %)	0,597

Improves oral health	0 (0 %)	9 (15,5 %)	7 (9 %)	4 (5,9 %)	5 (12,8 %)	25 (10,2 %)	0,413
I don't know	1 (33,3 %)	4 (6,9 %)	7 (9 %)	10 (14,7 %)	2 (5,2 %)	24 (9,7 %)	0,239
Total	3 (100 %)	58 (100 %	78 (100 %)	68 (100 %)	39 (100 %)	246 (100 %)	

15. What side effects do you think you may experience if you use antibiotics? You can choose more than one option

Variable	Frequency and percentage						*P-value*
Nausea or vomiting	0 (0 %)	7 (12,1 %)	7 (9 %)	23 (33,8 %)	12 (30,8 %)	49 (19,9%)	**0,001**
Diarrhea	0 (0 %)	7 (12,1 %)	18 (23,1 %)	25 (36,8 %)	18 (46,2 %)	68 (27,6 %)	**0,001**
Fever	1 (33,3%)	5 (8,6 %)	3 (3,8 %)	5 (7,4 %)	8 (20,5 %)	22 (8,9 %)	**0,023**
Fungal infections	1 (33,3 %)	5 (8,6 %)	12 (15,4 %)	16 (23,5 %)	14 (35,9 %)	48 (19,5 %)	**0,011**
Allergic reaction	0 (0%)	9 (15,5 %)	14 (17,9 %)	21 (30,9%)	16 (41 %)	60 (24,4 %)	**0,013**
None of the above	0 (0%)	13 (22,4 %)	14 (17,9 %)	4 (5,9 %)	4 (10,3 %)	35 (14,2 %)	0,062
I don't know	2 (66,7 %)	27 (46,6 %)	34 (43,6 %)	20 (29,4 %)	9 (23,1 %)	92 (37,4 %)	**0,045**
Total	3 (100 %)	58 (100 %	78 (100 %)	68 (100 %)	39 (100 %)	246 (100 %)	

16. When you take antibiotics, for how long do you take them

Variable	Frequency and percentage						*P-value*
1 day	0 (0 %)	0 (0 %)	0 (0 %)	0 (0 %)	1 (2,6 %)	1 (0,4 %)	0
2 days	1 (33,3 %)	0 (0 %)	3 (3,8 %)	1 (1,5 %)	1 (2,6 %)	6 (2,4 %)	
3-5 days	0 (0 %)	9 (15,5 %)	21 (26,9 %)	17 (25,4 %)	8 (20,5 %)	55 (22,4 %)	
7 days	0 (0 %)	44 (75,9 %)	47 (60,3 %)	38 (56,7 %)	26 (66,7 %)	155 (63,3 %)	
2 weeks or more	1 (33,3 %)	1 (1,7 %)	1 (1,3%)	0 (0 %)	0 (0 %)	3 (1,2 %)	
Other	1 (33,3 %)	4 (6,9 %)	6 (7,7 %)	11 (16,4 %)	3 (7,7 %)	25 (10,2 %)	
Total	3 (100 %)	58 (100 %)	78 (100 %)	68 (100 %)	39 (100 %)	245 (100 %)	

**17. You have knowledge of antibiotic resistance**							
Variable	Frequency and percentage						*P-value*
Yes	0 (0 %)	8 (13,8 %)	18 (23,4 %)	17 (25 %)	11 (28,2 %)	54 (22 %)	**0,012**
Yes. I think it is an issue of global health importance	0 (0 %)	17 (29,3 %)	19 (24,7 %)	28 (41,2%)	18 (46,2 %)	82 (33,5%)	
No	3 (100 %)	33 (56,9 %)	40 (51,9 %)	23 (33,8 %)	10 (25,6 %)	109 (44,5 %)	
Total	3 (100 %)	58 (100 %)	78 (100 %)	68 (100 %)	39 (100 %)	245 (100 %)	
**18. Have you ever had any teeth extracted?**							
Variable	Frequency and percentage						*P-value*
Yes	2 (66,7 %)	55 (94,8 %)	67 (85,9 %)	61 (89,7 %)	27 (69,2 %)	212 (86,2 %)	**0,005**
No	1 (33,3 %)	3 (5,2%)	11 (14,1%)	7 (10,3 %)	12 (30,8 %)	34 (13,8 %)	
Total	3 (100 %)	58 (100 %)	78 (100 %)	68 (100 %)	39 (100 %)	246 (100 %)	
**19. Have you ever had any more complicated oral surgery?**							
Variable	Frequency and percentage						*P-value*
Extraction of traumatic tooth (fractured tooth, tooth associated with a cyst, enclosed tooth)	0 (0 %)	17 (29,3%)	17 (21,8 %)	10 (14,7 %)	6 (15,4 %)	50 (20,3 %)	0,225
Sutura	0 (0 %)	11 (19 %)	11 (14,1 %)	8 (11,8 %)	1 (2,6 %)	31 (12,6 %)	0,175
Bone regeneration	0 (0 %)	3 (5,2%)	3 (3,8 %)	3 (4,4 %)	0 (0 %)	9 (3,7 %)	0,719
Others	0 (0 %)	2 (3,4 %)	7 (9 %)	10 (14,7 %)	3 (7,7 %)	22 (8,9 %)	0,259
No	3 (100 %)	32 (55,2 %)	46 (59 %)	40 (58,8 %)	29 (74,4%)	150 (61 %)	0,204
Total	3 (100 %)	58 (100 %	78 (100 %)	68 (100 %)	39 (100 %)	246 (100 %)	
**20. Before extracting the tooth, do you think it is necessary to take antibiotics?**							
Variable	Frequency and percentage						*P-value*

Yes	0 (0 %)	12 (20,7 %)	8 (10,3 %)	6 (8,8 %)	5 (12,8 %)	31 (12,6 %)	0,364
No	2 (66,7 %)	18 (31 %)	35 (44,9 %)	32 (47,1%)	14 (35,9 %)	101 (41,1 %)	
On some occasions	0 (0 %)	23 (39,7 %)	25 (32,1 %)	26 (38,2 %)	15 (38,5 %)	89 (36,2 %)	
I don't know	1 (33,3 %)	5 (8,6 %)	10 (12,8 %)	4 (5,9 %)	5 (12,8 %)	25 (10,2 %)	
Total	3 (100 %)	58 (100 %)	78 (100 %)	68 (100 %)	39 (100 %)	246 (100 %)	

21. After extracting the tooth, do you think it is necessary to take antibiotics?

Variable	Frequency and percentage						*P-value*
Yes	2 (66,7 %)	7 (12,1 %)	19 (24,4 %)	14 (20,6 %)	8 (20,5 %)	50 (20,3 %)	0,603
No	0 (0 %)	18 (31 %)	17 (21,8 %)	15 (22,1 %)	7 (17,9 %)	57 (23,2 %)	
On some occasions	1 (33,3 %)	29 (50 %)	34 (43,6 %)	34 (50 %)	20 (51,3 %)	118 (48 %)	
I don't know	0 (0 %)	4 (6,9 %)	8 (10,3 %)	5 (7,4 %)	4 (10,3 %)	21 (8,5 %)	
Total	3 (100 %)	58 (100 %)	78 (100 %)	68 (100 %)	39 (100 %)	246 (100 %)	

22. Do you think that antibiotics would be indicated for patients with diseases that are more likely to cause complications after tooth extrations?

Variable	Frequency and percentage						*P-value*
Yes	2 (66,7 %)	22 (37,9 %)	38 (48,7 %)	29 (42,6 %)	11 (28,2 %)	102 (41,5 %)	0,073
No	1 (33,3 %)	11 (19 %)	12 (15,4 %)	9 (13,2 %)	2 (5,1 %)	35 (14,2 %)	
On some occasions	0 (0 %)	25 (43,1 %)	28 (35,9 %)	30 (44,1 %)	26 (66,7 %)	109 (44,3 %)	
Total	3 (100 %)	58 (100 %)	78 (100 %)	68 (100 %)	39 (100 %)	246 (100 %)	

23. Have you ever self-medicated with antibiotics before or after having a tooth removed?

Variable	Frequency and percentage						*P-value*
Yes	2 (66,7 %)	13 (22,4 %)	11 (14,1 %)	7 (10,3 %)	5 (12,8 %)	38 (15,4 %)	**0,043**
No	1	45	67	61	34	208	

	(33,3 %)	(77,6 %)	(85,9 %)	(89,7 %)	(87,2 %)	(84,6 %)	
Total	3 (100 %)	58 (100 %)	78 (100 %)	68 (100 %)	39 (100 %)	246 (100 %)	
24. Have you ever had any implant surgery that you consider simple?							
Variable	Frequency and percentage						*P-value*
Yes	0 (0 %)	5 (8,6 %)	10 (12,8 %)	9 (13,2 %)	3 (7,7 %)	27 (11 %)	0,780
No	3 (100 %)	53 (91,4 %)	68 (87,2 %)	59 (86,8 %)	36 (92,3 %)	219 (89%)	
Total	3 (100 %)	58 (100 %)	78 (100 %)	68 (100 %)	39 (100 %)	246 (100 %)	
25. Have you had implant surgery that you consider complex?							
Variable	Frequency and percentage						*P-value*
Yes. Orthopedic rehabilitation-related surgery	0 (0 %)	0 (0 %)	0 (0 %)	1 (1,5 %)	0 (0 %)	1 (0,4 %)	0,622
Yes, surgery related to bone regeneration.	0 (0 %)	0 (0 %)	0 (0 %)	2 (2,9 %)	0 (0 %)	2 (0,8 %)	0,260
Yes. Implant-related surgery	0 (0 %)	0 (0 %)	1 (1,3 %)	3 (4,4 %)	0 (0 %)	4 (1,6 %)	0,286
Yes. Surgery related to molar exodontics	0 (0 %)	1 (1,7 %)	0 (0 %)	0 (0 %)	0 (0 %)	1 (0,4 %)	0,516
Yes. Surgery related to failed implants	0 (0 %)	0 (0 %)	0 (0 %)	1 (1,5 %)	0 (0 %)	1 (0,4 %)	0,622
No	3 (100 %)	56 (96,6 %)	76 (97,4 %)	61 (89,7 %)	39 (100 %)	235 (95,5 %)	0,086
Total	3 (100 %)	58 (100 %)	78 (100 %)	68 (100 %)	39 (100 %)	246 (100 %)	

Table S4. Contingency table. Group 2 - 3 (Students and professors)						
**AGE**						
	**Students**	**Undergraduate professor**	**Postgraduate professor**	**Undergraduate and Postgraduate professor**	**Total**	
Variable	Frecuency and percentage					*P-value*
20 - 25	53 (67,1 %)	0 (0 %)	0 (0 %)	0 (0 %)	53 (41,1 %)	0
25 - 30	20 (25,3 %)	0 (0 %)	1 (20 %)	1 (4,8 %)	22 (17,1 %)	
30 - 35	3 (3,8 %)	3 (12,5 %)	3 (60 %)	6 (28,6 %)	15 (11,6 %)	
35 - 45	2 (2,5 %)	4 (16,7 %)	1 (20 %)	5 (23,8 %)	12 (9,3 %)	
45 - 55	1 (1,3 %)	9 (37,5 %)	0 (0 %)	3 (14,3 %)	13 (10,1 %)	
55 - 70	0 (0 %)	8 (33,3 %)	0 (0 %)	6 (28,6 %)	14 (10,9 %)	
Total	79 (100 %)	24 (100 %)	5 (100 %)	21 (100 %)	129 (100%)	
	**Students**	**Undergraduate professor**	**Postgraduate professor**	**Undergraduate and Postgraduate professor**	**Total**	
**GENDER**						
	**Gender**				Total	
Variable	Frecuency and percentage					*P-value*
Male	18 (22,8 %)	11 (45,8 %)	1 (20 %)	11 (52,4 %)	41 (31,8 %)	**0,023**
Female	61 (77,2 %)	13 (54,2 %)	4 (80 %)	10 (47,6 %)	88 (68,2 %)	
Total	79 (100 %)	24 (100 %)	5 (100 %)	21 (100 %)	129 (100%)	

Table S5. Contingency table. Group 3 (Professors).										
**SPECIALITY**										
	**I am a professor, but I do not have any additional qualifications**	**Yes. Master's or postgraduate degree in Surgery, Oral Medicine, Implantology or Oncology Patients**	**Yes. Master's or postgraduate degree in Endodontics**	**Yes. Master's or postgraduate degree in Pediatric Dentistry or Gerodontology**	**Yes. Master's or postgraduate degree in Orthodontics**	**Yes. Master's or postgraduate degree in Prosthetics or Integrated Adult Dentistry**	**Yes. Master's or postgraduate degree in Periodontics**	**Yes. Master's or postgraduate degree in other specialties**	**Total**	
6. How many root canals do you perform in a week?										
Variable	Frecuency and percentage									*P-value*
I don't do that kind of treatment	0 (0 %)	7 (58,3 %)	0 (0 %)	2 (50 %)	2 (40 %)	4 (30,8 %)	3 (75 %)	10 (71,4 %)	24 (48 %)	0, 289
1 or none	0 (0 %)	0 (0 %)	1 (20 %)	0 (0 %)	1 (20 %)	3 (23,1 %)	1 (25 %)	0 (0 %)	6 (12 %)	
Between 2–4	1 (50 %)	3 (25 %)	1 (20 %)	2 (50 %)	2 (40 %)	5 (38,5 %)	0 (0 %)	2 (14,3 %)	12 (24 %)	
Between 4–8	0 (0 %)	1 (8,3 %)	1 (20 %)	0 (0 %)	0 (0 %)	0 (0 %)	0 (0 %)	1 (7,1 %)	3 (6 %)	
More than 8	1 (50 %)	1 (8,3 %)	2 (40 %)	0 (0 %)	0 (0 %)	1 (7,7 %)	2 (14,3 %)	1 (7,1 %)	5 (10 %)	
Total	2 (100 %)	12 (100 %)	5 (100 %)	4 (100 %)	5 (100 %)	13 (100 %)	4 (100 %)	14 (100 %)	-	
7. If systemic antibiotic administration (oral) is indicated, which of the following antibiotics would you choose for treating odontogenic infections in an adult without allergies?										
Variable	Frecuency and percentage									*P-value*
Amoxicillin 500mg	0 (0 %)	1 (8,3 %)	1 (20 %)	1 (25 %)	2 (40 %)	0 (0 %)	1 (25 %)	4 (28,6 %)	8 (16 %)	0,433
Amoxicillin 750mg	1 (50 %)	10 (83,3 %)	2 (40 %)	3 (75 %)	3 (60 %)	8 (61,5 %)	3 (75 %)	8 (57,1 %)	32 (64 %)	
Amoxicillin 1g	0 (0 %)	0 (0 %)	1 (20 %)	0 (0 %)	0 (0 %)	1 (7,7 %)	0 (0 %)	0 (0 %)	2 (4 %)	

Amoxicillin / Clavulanic acid 250/82,5	0 (0 %)	0 (0 %)	0 (0 %)	0 (0 %)	0 (0 %)	0 (0 %)	0 (0 %)	1 (7,1 %)	1 (2 %)	
Amoxicillin / Clavulanic acid 500/125	0 (0 %)	0 (0 %)	1 (20 %)	0 (0 %)	0 (0 %)	0 (0 %)	0 (0 %)	0 (0 %)	1 (2 %)	
Amoxicillin / Clavulanic acid 875/125	1 (50 %)	1 (8,3 %)	0 (0 %)	0 (0 %)	0 (0 %)	4 (30,8 %)	0 (0 %)	1 (7,1 %)	6 (12 %)	
Clindamycin 300mg	0 (0 %)	0 (0 %)	0 (0 %)	0 (0 %)	0 (0 %)	0 (0 %)	0 (0 %)	0 (0 %)	0 (0 %)	
Clindamycin 600mg	0 (0 %)	0 (0 %)	0 (0 %)	0 (0 %)	0 (0 %)	0 (0 %)	0 (0 %)	0 (0 %)	0 (0 %)	
Azithromycin 150mg	0 (0 %)	0 (0 %)	0 (0 %)	0 (0 %)	0 (0 %)	0 (0 %)	0 (0 %)	0 (0 %)	0 (0 %)	
Azithromycin 200mg	0 (0 %)	0 (0 %)	0 (0 %)	0 (0 %)	0 (0 %)	0 (0 %)	0 (0 %)	0 (0 %)	0 (0 %)	
Azithromycin 250mg	0 (0 %)	0 (0 %)	0 (0 %)	0 (0 %)	0 (0 %)	0 (0 %)	0 (0 %)	0 (0 %)	0 (0 %)	
Azithromycin 500mg	0 (0 %)	0 (0 %)	0 (0 %)	0 (0 %)	0 (0 %)	0 (0 %)	0 (0 %)	0 (0 %)	0 (0 %)	
Azithromycin 1g	0 (0 %)	0 (0 %)	0 (0 %)	0 (0 %)	0 (0 %)	0 (0 %)	0 (0 %)	0 (0 %)	0 (0 %)	
Metronidazole	0 (0 %)	0 (0 %)	0 (0 %)	0 (0 %)	0 (0 %)	0 (0 %)	0 (0 %)	0 (0 %)	0 (0 %)	
Erythromycin	0 (0 %)	0 (0 %)	0 (0 %)	0 (0 %)	0 (0 %)	0 (0 %)	0 (0 %)	0 (0 %)	0 (0 %)	
Lincosamine	0 (0 %)	0 (0 %)	0 (0 %)	0 (0 %)	0 (0 %)	0 (0 %)	0 (0 %)	0 (0 %)	0 (0 %)	
Total	2 (100 %)	12 (100 %)	5 (100 %)	4 (100 %)	5 (100 %)	13 (100 %)	4 (100 %)	14 (100 %)	-	
8. How long would you prescribe the antibiotic?										
Variable	Frecuency and percentage									*P-value*
1 day	0 (0 %)	0 (0 %)	0 (0 %)	0 (0 %)	0 (0 %)	0 (0 %)	0 (0 %)	0 (0 %)	0 (0 %)	0,324
2 days	0 (0 %)	0 (0 %)	0 (0 %)	0 (0 %)	0 (0 %)	0 (0 %)	0 (0 %)	0 (0 %)	0 (0 %)	
3–5 days	0 (0 %)	4 (33 ,3 %)	2 (40 %)	2 (50 %)	2 (40 %)	2 (15,4 %)	0 (0 %))	2 (14,3 %)	10 (20 %)	
7 days	2 (100 %)	8 (66,7 %)	3 (60 %)	2 (50 %)	3 (60 %)	11 (84,6 %)	4 (100 %)	12 (85,7 %)	40 (80 %)	
10 days	0 (0 %)	0 (0 %))	0 (0 %)	0 (0 %)	0 (0 %)	0 (0 %)	0 (0 %)	0 (0 %)	0 (0 %)	

Total	2 (100 %)	12 (100 %)	5 (100 %)	4 (100 %)	5 (100 %)	13 (100 %)	4 (100 %)	14 (100 %)	-	

9. If systemic administration of antibiotics (oral route) is indicated, which of these antibiotics would you choose for the odontogenic infectious treatment of an adult with penicillin allergy?

Variable	Frecuency and percentage									*P-value*
Clindamycin 300mg	1 (50 %)	5 (41,7 %)	3 (60 %)	2 (50 %)	3 (60 %)	9 (69,2 %)	1 (25 %)	6 (42,9 %)	25 (50 %)	**0,016**
Clindamycin 600mg	1 (50 %)	1 (8,3 %)	1 (20 %)	0 (0 %)	0 (0 %)	2 (15,4 %)	1 (25 %)	1 (7,1 %)	6 (12 %)	
Azithromycin 150mg	0 (0 %)	0 (0 %)	0 (0 %)	0 (0 %)	0 (0 %)	0 (0 %)	0 (0 %)	0 (0 %)	0 (0 %)	
Azithromycin 200mg	0 (0 %)	0 (0 %)	0 (0 %)	0 (0 %)	0 (0 %)	0 (0 %)	0 (0 %)	0 (0 %)	0 (0 %)	
Azithromycin 250mg	0 (0 %)	0 (0 %)	0 (0 %)	0 (0 %)	0 (0 %)	0 (0 %)	0 (0 %)	0 (0 %)	0 (0 %)	
Azithromycin 500mg	0 (0 %)	6 (50 %)	0 (0 %)	0 (0 %)	1 (20 %)	1 (7,7 %)	2 (50 %)	6 (42,9 %)	15 (30 %)	
Azithromycin 1g	0 (0 %)	0 (0 %)	1 (20 %)	0 (0 %)	0 (0 %)	0 (0 %)	0 (0 %)	0 (0 %)	1 (2 %)	
Metronidazole	0 (0 %)	0 (0 %)	0 (0 %)	0 (0 %)	0 (0 %)	0 (0 %)	0 (0 %)	0 (0 %)	0 (0 %)	
Erythromycin	0 (0 %)	0 (0 %)	0 (0 %)	2 (50 %)	1 (20 %)	0 (0 %)	0 (0 %)	1 (7,1 %)	2 (4 %)	
Lincosamine	0 (0 %)	0 (0 %)	0 (0 %)	0 (0 %)	0 (0 %)	1 (7,1 %)	0 (0 %)	0 (0 %)	1 (2 %)	
Total	2 (100 %)	12 (100 %)	5 (100 %)	4 (100 %)	5 (100 %)	13 (100 %)	4 (100 %)	14 (100 %)	-	

Table S6. Contingency table. Group 3 (Professors).										
SPECIALITY										
	**I am a professor, but I do not have any additional qualifications**	**Yes. Master's or postgraduate degree in Surgery, Oral Medicine, Implantology or Oncology Patients**	**Yes. Master's or postgraduate degree in Endodontics**	**Yes. Master's or postgraduate degree in Pediatric Dentistry or Gerodontology**	**Yes. Master's or postgraduate degree in Orthodontics**	**Yes. Master's or postgraduate degree in Prosthetics or Integrated Adult Dentistry**	**Yes. Master's or postgraduate degree in Periodontics**	**Yes. Master's or postgraduate degree in other specialties**	**Total**	**P-value**
10. In which of these clinical situations do you think the use of systemic (oral) antibiotics would be indicated? You may tick more than one.										
Variable	Frecuency and percentage									*P-value*
Symptomatic irreversible pulpitis, with moderate or severe preoperative symptoms	0 (0 %)	1 (9,1 %)	1 (25 %)	0 (0 %)	1 (25 %)	0 (0 %)	1 (25 %)	6 (42,9 %)	10 (20 %)	0,731
Irreversible pulpitis with periapical involvement with moderate or severe preoperative symptoms	1 (50 %)	2 (18,2 %)	0 (0 %)	2 (66,7 %)	3 (75%)	3 (23,1 %)	1 (25 %)	3 21,4 %)	12 (24 %)	
Pulp necrosis with asymptomatic apical periodontitis, without abscess with mild or no symptoms	0 (0 %)	1 (9,1 %)	1 (25 %)	0 (0 %)	0 (0 %)	2 (15,4 %)	1 (25 %)	2 (14,3 %)	6 (12 %)	
Pulp necrosis with symptomatic apical periodontitis, without abscess with moderate or severe symptoms	2 (100 %)	2 (18,2 %)	2 (50 %)	1 (33,3 %)	2 (50 %)	5 (38,5 %)	2 (50 %)	5 (35,7 %)	19 (38 %)	
Pulp necrosis with asymptomatic apical periodontitis, fistulous tract present, mild or no symptoms	0 (0 %)	4 (36,4 %)	1 (25 %)	0 (0 %)	1 (25 %)	4 (30,8 %)	2 (50 %)	4 (28,6 %)	15 (30 %)	
Pulp necrosis with symptomatic apical	2 (100 %)	11 (100 %)	4 (100 %)	3 (100 %)	4 (100 %)	12 (92,3 %)	4 (100 %)	14 (100 %)	46 (92 %)	

periodontitis, abscess, moderate or severe symptoms										
Total	2 (100 %)	11 (100 %)	4 (100 %)	3 (100 %)	4 (100 %)	13 (100 %)	4 (100 %)	14 (100 %)	-	
11. If antibiotic prophylaxis is indicated, which protocol do you consider most appropiate for a patient with no penicillin allergies?										
Variable	Frecuency and percentage									*P-value*
Amoxicillin orally 2gr 1 hour before	2 (100 %)	12 (100 %)	5 (100 %)	4 (100 %)	5 (100 %)	13 (100 %)	4 (100 %)	14 (100 %)	50 (100 %)	0
Amoxicillin orally 1gr 1 hour before	0 (0 %)	0 (0 %)	0 (0 %)	0 (0 %)	0 (0 %)	0 (0 %)	0 (0 %)	0 (0 %)	0 (0 %)	
Amoxicillin orally 1gr 1 hour before and 1 hour after	0 (0 %)	0 (0 %)	0 (0 %)	0 (0 %)	0 (0 %)	0 (0 %)	0 (0 %)	0 (0 %)	0 (0 %)	
Total	2 (100 %)	12 (100 %)	5 (100 %)	4 (100 %)	5 (100 %)	13 (100 %)	4 (100 %)	14 (100 %)	-	
12. In the following cases, in which do you consider antibiotic prophylaxis to be indicated? (You can check more than one option):										
Variable	Frecuency and percentage									*P-value*
Immunocompromised or medically compromised patient	1 (50 %)	4 (33,3 %)	5 (100 %)	1 (25 %)	1 (20 %)	8 (61,5 %)	4 (100 %)	11 (78,6 %)	30 (60 %)	**0,002**
Patient taking oral bisphosphonates	0 (0 %)	1 (8,3 %)	1 (20 %)	0 (0 %)	1 (20 %)	5 (38,5 %)	1 (25 %)	7 (50 %)	14 (28 %)	
Patient taking bisphosphonates intravenously	2 (100 %)	1 (8,3 %)	1 (20 %)	1 (25 %)	1 (20 %)	6 (46,2 %)	2 (50 %)	8 (57,1 %)	20 (40 %)	
Patient at risk for bacterial infective endocarditis	2 (100 %)	12 (100 %)	4 (80 %)	4 (100 %)	5 (100 %)	12 (92,3 %)	3 (75 %)	14 (100 %)	48 (96 %)	
Patient with head and/or neck cancer associated with radiotherapy.	1 (50 %)	2 (16,7 %)	1 (20 %)	1 (25 %)	2 (40 %)	2 (15,4 %)	0 (0 %)	7 (50 %)	14 (28 %)	
Patient with joint prostheses	0 (0 %)	0 (0 %)	0 (0 %)	1 (25 %)	2 (40 %)	3 (23,1 %)	3 (75 %)	4 (28,6 %)	12 (24 %)	
Total	2 (100 %)	12 (100 %)	5 (100 %)	4 (100 %)	5 (100 %)	13 (100 %)	4 (100 %)	14 (100 %)	-	
13. How many extractions do you perfom in a week?										
Variable	Frecuency and percentage									*P-value*

I do not perform these treatments	1 (50 %)	0 (0 %)	2 (40 %)	2 (50 %)	3 (60 %)	0 (0 %)	0 (0 %)	2 (14,3 %)	7 (14 %)	
Between 1 or none	0 (0 %)	2 (16, 7 %)	1 (20 %)	0 (0 %)	0 (0 %)	1 (7,7 %)	2 (50 %)	2 (14,3 %)	7 (14 %)	
Between 2-4	1 (50 %)	3 (25 %)	1 (20 %)	0 (0 %)	0 (0 %)	9 (69,2 %)	1 (25 %)	3 (21,4 %)	15 (30 %)	
Between 4-8	0 (0 %)	2 (16,7 %)	1 (20 %)	0 (0 %)	0 (0 %)	2 (15,4 %)	0 (0 %)	0 (0 %)	5 (10 %)	0,006
More tan 8	0 (0 %)	5 (41,7 %)	0 (0 %)	2 (50 %)	2 (40 %)	1 (7,7 %)	1 (25 %)	7 (50 %)	16 (32 %)	
Total	2 (100 %)	12 (100 %)	5 (100 %)	4 (100 %)	5 (100 %)	13 (100 %)	4 (100 %)	14 (100 %)	-	

14. In which of these clinical situations do you consider systemic antibiotic use to be indicated? (You may select more than one):

Variable	Frecuency and percentage									*P-value*
Simple extraction in a healthy patient	0 (0 %)	0 (0 %)	0 (0 %)	0 (0 %)	0 (0 %)	0 (0 %)	0 (0 %)	0 (0 %)	0 (0 %)	
Simple extraction in immunocompromised patients.	1 (50 %)	4 (33,3 %)	4 (80 %)	2 (50 %)	3 (60 %)	6 (46,2 %)	3 (75 %)	10 (76,9 %)	29 (58 %)	
Surgical extraction (odontosection and/or ostectomy, either of included teeth or third molars) in healthy patients.	1 (50 %)	8 (66,7 %)	2 (40 %)	2 (50 %)	2 (40 %)	5 (38,5 %)	0 (0 %)	6 (46,2 %)	23 (46 %)	
Surgical extraction (odontosection and/or ostectomy, either of included teeth or third molars) in immunocompromised patients.	2 (100 %)	12 (100 %)	4 (80 %)	4 (100 %)	5 (100 %)	12 (92,3 %)	3 (75 %)	13 (100 %)	47 (94 %)	0,177
Bone regeneration	2 (100 %)	11 (91,7 %)	3 (60 %)	1 (25 %)	2 (40 %)	11 (84,6 %)	2 (50 %)	9 (69,2 %)	36 (72 %)	
Implant placement	1 (50 %)	8 (66,7 %)	2 (40 %)	1 (25 %)	2 (40 %)	10 (76,90 %)	2 (50 %)	6 (46,2 %)	27 (54 %)	
Bone regeneration with implant placement.	2 (100 %)	10 (83,3 %)	4 (80 %)	2 (50 %)	3 (60 %)	11 (84,6 %)	3 (75 %)	9 (69,2 %)	38 (76 %)	
Maxillary sinus elevation	1 (50 %)	11 (91,7 %)	4 (80 %)	2 (50 %)	3 (60 %)	12 (92,3 %)	2 (50 %)	9 (69,2 %)	38 (76 %)	
Total	2 (100 %)	12 (100 %)	5 (100 %)	4 (100 %)	5 (100 %)	13 (100 %)	4 (100 %)	14 (100 %)	-	

Table S7. Contingency table. Group 2 - 3 (Students - Professors).				
**STUDENTS OR PROFESSORS**				
	**Students**	**Professors**	**Total**	**P-value**
6. How many root canals do you perform in a week?				
Variable	Frecuency and percentage			*P-value*
I don't do that kind of treatment	8 (10,1 %)	24 (48 %)	32 (24, 8 %)	0
1 or none	67 (84,8 %)	6 (12 %)	73 (56,6 %)	
Between 2–4	4 (5,1 %)	12 (24%)	16 (12,4 %)	
Between 4–8	0 (0 %)	3 (6 %)	3 (2,3 %)	
More than 8	0 (0 %)	5 (10 %)	5 (3,9 %)	
Total	79 (100 %)	50 (100 %)	129 (100 %)	
7. If systemic antibiotic administration (oral) is indicated, which of the following antibiotics would you choose for treating odontogenic infections in an adult without allergies?				
Variable	Frecuency and percentage			*P-value*
Amoxicillin 500mg	11 (13,9 %)	8 (16 %)	19 (14,7 %)	0,062
Amoxicillin 750mg	63 (79,7 %)	32 (64 %)	95 (73,6 %)	
Amoxicillin 1g	1 (1,3 %)	2 (4 %)	3 (2,3 %)	
Amoxicillin / Clavulanic acid 250/82,5	0 (0 %)	1 (2 %)	1 (0,8 %)	
Amoxicillin / Clavulanic acid 500/125	3 (3,8 %)	1 (2 %)	4 (3.1 %)	
Amoxicillin / Clavulanic acid 875/125	1 (1,3 %)	6 (12 %)	7 (5,4 %)	
Clindamycin 300mg	0 (0 %)	0 (0 %)	0 (0 %)	

Clindamycin 600mg	0 (0 %)	0 (0 %)	0 (0 %)	
Azithromycin 150mg	0 (0 %)	0 (0 %)	0 (0 %)	
Azithromycin 200mg	0 (0 %)	0 (0 %)	0 (0 %)	
Azithromycin 250mg	0 (0 %)	0 (0 %)	0 (0 %)	
Azithromycin 500mg	0 (0 %)	0 (0 %)	0 (0 %)	
Azithromycin 1g	0 (0 %)	0 (0 %)	0 (0 %)	
Metronidazole	0 (0 %)	0 (0 %)	0 (0 %)	
Erythromycin	0 (0 %)	0 (0 %)	0 (0 %)	
Lincosamine	0 (0 %)	0 (0 %)	0 (0 %)	
Total	79 (100 %)	50 (100 %)	129 (100 %)	
8. How long would you prescribe the antibiotic?				
Variable	Frecuency and percentage			*P-value*
1 day	0 (0 %)	0 (0 %)	0 (0 %)	0,088
2 days	0 (0 %)	0 (0 %)	0 (0 %)	
3–5 days	6 (7,6 %)	10 (20 %)	16 (12,4 %)	
7 days	72 (91,1 %)	40 (80 %)	112 (86,8 %)	
10 days	1 (1,3 %)	0 (0 %)	1 (0,8 %)	
Total	79 (100 %)	50 (100 %)	129 (100 %)	
9. If systemic administration of antibiotics (oral route) is indicated, which of these antibiotics would you choose for the odontogenic infectious treatment of an adult with penicillin allergy?				
Variable	Frecuency and percentage			*P-value*
Clindamycin 300mg	23 (29,1 %)	25 (50 %)	48 (37,2 %)	**0,002**

Clindamycin 600mg	34<br>(43 %)	6<br>(12 %)	40<br>(31 %)	
Azithromycin 150mg	0<br>(0 %)	0<br>(0 %)	0<br>(0 %)	
Azithromycin 200mg	0<br>(0 %)	0<br>(0 %)	0<br>(0 %)	
Azithromycin 250mg	2<br>(2,5 %)	0<br>(0 %)	2<br>(1,6 %)	
Azithromycin 500mg	20<br>(25, 3 %)	15<br>(30 %)	35<br>(27,1 %)	
Azithromycin 1g	0<br>(0 %)	1<br>(2,0 %)	1<br>(0,8 %)	
Metronidazole	0<br>(0 %)	0<br>(0 %)	0<br>(0 %)	
Erythromycin	0<br>(0 %)	2<br>(4 %)	0<br>(0 %)	
Lincosamine	0<br>(0 %)	1<br>(2 %)	1<br>(0,8 %)	
Total	79<br>(100 %)	50<br>(100 %)	129<br>(100 %)	

Table S8. Contingency table. Group 2 - 3 (Students - Professors).				
**STUDENTS OR PROFESSORS**				
	**Students**	**Professors**	**Total**	**P-value**
10. In which of these clinical situations do you think the use of systemic (oral) antibiotics would be indicated? You may tick more than one.				
Variable	Frecuency and percentage			*P-value*
Symptomatic irreversible pulpitis, with moderate or severe preoperative symptoms	8 (10,1 %)	10 (20 %)	18 (14 %)	0,115
Irreversible pulpitis with periapical involvement with moderate or severe preoperative symptoms	26 (32,9 %)	12 (24 %)	38 (29,5 %)	0,279
Pulp necrosis with asymptomatic apical periodontitis, without abscess with mild or no symptoms	2 (2,5 %)	6 (12 %)	8 (6,2 %)	**0,030**
Pulp necrosis with symptomatic apical periodontitis, without abscess with moderate or severe symptoms	18 (22,8 %)	19 (38 %)	37 (28,7 %)	0,063
Pulp necrosis with asymptomatic apical periodontitis, fistulous tract present, mild or no symptoms	21 (26.6 %)	15 (30 %)	36 (27,9 %)	0,673
Pulp necrosis with symptomatic apical periodontitis, abscess, moderate or severe symptoms	72 (91,1 %)	46 (92 %)	118 (91,5 %)	0,865
Total	79 (100 %)	50 (100 %)	129 (100 %)	
11. If antibiotic prophylaxis is indicated, which protocol do you consider most appropiate for a patient with no penicillin allergies?				
Variable	Frecuency and percentage			*P-value*
Amoxicillin orally 2gr 1 hour before	71 (89,9 %)	50 (100 %)	121 (93,8 %)	**0,020**
Amoxicillin orally 1gr 1 hour before	8 (10,1 %)	0 (0 %)	8 (6,2 %)	
Amoxicillin orally 1gr 1 hour before and 1 hour after	0 (0 %)	0 (0 %)	0 (0 %)	
Total	79 (100 %)	50 (100 %)	129 (100 %)	

12. In the following cases, in which do you consider antibiotic prophylaxis to be indicated? (You can check more than one option):				
Variable	Frecuency and percentage			*P-value*
Immunocompromised or medically compromised patient	67 (84,8 %)	30 (60 %)	97 (75,2 %)	**0,001**
Patient taking oral bisphosphonates	10 (12,7 %)	14 (28 %)	24 (18,6 %)	**0,029**
Patient taking bisphosphonates intravenously	36 (45,6 %)	20 (40 %)	56 (43,4 %)	0,534
Patient at risk for bacterial infective endocarditis	77 (97,5 %)	48 (96 %)	125 (96,9 %)	0,639
Patient with head and/or neck cancer associated with radiotherapy.	16 (20,3 %)	14 (28 %)	30 (23,3 %)	0,310
Patient with joint prostheses	29 (36,7 %)	12 (24 %)	41 (31,8 %)	0,131
Total	79 (100 %)	50 (100 %)	129 (100 %)	

13. How many extractions do you perfom in a week?				
Variable	Frecuency and percentage			*P-value*
I do not perform these treatments	2 (2,5 %)	7 (14 %)	9 (7 %)	0
Between 1 or none	61 (77,2 %)	7 (140 %)	68 (52,7 %)	
Between 2-4	16 (20,3 %)	15 (30 %)	31 (24 %)	
Between 4-8	0 (0 %)	5 (10 %)	5 (3,9 %)	
More tan 8	0 (0 %)	16 (32 %)	16 (14,4 %)	
Total	79 (100 %)	50 (100 %)	129 (100 %)	

14. In which of these clinical situations do you consider systemic antibiotic use to be indicated? (You may select more than one):				
Variable	Frecuency and percentage			*P-value*
Simple extraction in a healthy patient	0 (0 %)	0 (0 %)	0 (0 %)	-
Simple extraction in immunocompromised patients.	46 (58,2 %)	29 (58 %)	75 (58,1 %)	0,980
Surgical extraction (odontosection and/or ostectomy, either of included teeth or third molars) in healthy patients.	25 (31,6 %)	23 (46 %)	48 (37,2 %)	0,100

Surgical extraction (odontosection and/or ostectomy, either of included teeth or third molars) in immunocompromised patients.	72 (91,1 %)	47 (94 %)	119 (92,2 %)	0,554
Bone regeneration	42 (53,2 %)	36 (72 %)	78 (60,5 %)	0,033
Implant placement	17 (21,5 %)	27 (54 %)	44 (34,1 %)	0,000
Bone regeneration with implant placement.	42 (53,2 %)	38 (76 %)	80 (62 %)	0,009
Maxillary sinus elevation	38 (48,1 %)	38 (76 %)	76 (58,9 %)	0,002
Total	79 (100 %)	50 (100 %)	129 (100 %)	
